# Mothers’ and fathers’ neural responses toward gender-stereotype violations by their own children

**DOI:** 10.1093/scan/nsae025

**Published:** 2024-03-29

**Authors:** Christel M Portengen, Anneloes L van Baar, Joyce J Endendijk

**Affiliations:** Child and Adolescent Studies, Utrecht University, Utrecht 3584 CS, The Netherlands; Child and Adolescent Studies, Utrecht University, Utrecht 3584 CS, The Netherlands; Child and Adolescent Studies, Utrecht University, Utrecht 3584 CS, The Netherlands

**Keywords:** gender stereotypes, own children, unknown children, electroencephalography, parents

## Abstract

Gender stereotypes facilitate people’s processing of social information by providing assumptions about expected behaviors and preferences. When gendered expectations are violated, people often respond negatively, both on a behavioral and neural level. Little is known about the impact of family kinship on the behavioral and neural reactions to gender-stereotype violations. Therefore, we examined whether parents show different responses when gender stereotypes are violated by their own children *vs* unknown children. The sample comprised 74 Dutch families with a father (*M*_age_ = 37.54), mother (*M*_age_ = 35.83), son, and daughter aged 3–6 years. Electroencephalography measurements were obtained while parents viewed pictures of their own and unknown children paired with toy or problem behavior words that violated or confirmed gender stereotypes. In half of the trials, parents evaluated the appropriateness of toy–gender and behavior–gender combinations. Parents showed stronger late positive potential amplitudes toward gender stereotype-violating behaviors by own children compared to unknown children. Moreover, parents’ P1 responses toward gender stereotype-violating child behaviors were stronger for boys than for girls and for parents who evaluated gender-stereotype violations as less appropriate than gender-stereotype confirmations. These findings indicated that gender-stereotype violations by parents’ own children are particularly salient and viewed as less appropriate than gender-stereotype confirmations.

## Introduction

In a complex social world, people quickly need to process information to understand what is happening and to adapt their behavior accordingly. Gender stereotypes facilitate fast processing of social information by providing assumptions about the expected behavior, roles, and characteristics of men and women (Darley and Gross, 1983). Gender stereotypes can simultaneously be descriptive and prescriptive (Koenig, 2018). The descriptive component of gender stereotypes refers to the beliefs that people have about what a man or woman normally does. The prescriptive component of stereotypes revolves around how a man or woman should behave. Violations of prescriptive gender stereotypes generally elicit strong negative responses in people (Rudman *et al*., 2012a, 2012b; Koenig, 2018; Sullivan *et al*., 2018).

In line with these findings, parents respond more negatively toward children who violate gender stereotypes (e.g. a boy playing with dolls) than children who adhere to gendered norms (e.g. a boy playing with cars) (Kane, 2006; Friedman *et al*., 2007; Endendijk *et al*., 2014). Also, parents expect children who do not adhere to traditional gender norms to be less well-adjusted later in life than children who do adhere to gender norms (Sandnabba and Ahlberg, 1999). To increase insight into the underlying mechanisms associated with people’s negative responses to gender-stereotype violations, social neuroscientists have identified several key brain areas that play a prominent role in the processing of stereotyped information (Stanley *et al*., 2008; Amodio, 2014). However, thus far, most of the studies that focused on parents’ responses to gender-stereotype violations have examined brain responses to the violation and confirmation of gendered expectations about children in general. Little is known about parents’ neural responses to gender-stereotype violations and confirmations when it concerns their own children. It is important to gain more knowledge on how parents process and respond to violations of gender stereotypes by their own children (compared to other children), since these processes might better explain gendered parenting behaviors with their own children than neural processing of other children’s behavior that violates gender stereotypes. In addition, comparing parents’ neural responses to own and other children allows for the investigation of the impact of familial kinship on the neural processing of gender-stereotype violations. Gaining information about parents’ neural processing of gendered behavior of their own children (*vs* other children) would moreover further the knowledge on the ecological validity of social neuroscientific research (Derks *et al*., 2013; Feldman, 2015).

The literature to date provides little clarity about whether parents would respond stronger, or less strong, to the violation of gender stereotypes when it concerns their own *vs* other children. Yet, parents and their children have strong affectional ties. Not surprisingly, research shows that parents’ own children evoke stronger physiological (Wiesenfeld and Klorman, 1978; Wiesenfeld *et al*., 1981), hormonal (Feldman, 2015) and neural (Doi and Shinohara, 2012; Bornstein *et al*., 2013) responses than unknown children. Based on the social judgment literature (Krueger and Rothbart, 1988) and research on parental cognitions, two contrasting hypotheses can be formulated with regard to parents’ neural responses toward gender-stereotype violations of own *vs* unknown children.

First, individuating information (i.e., information that makes a person an individual instead of a group member) and familiarity with a person’s character and preferences have the ability to override the impact of stereotyped expectations of people and alter neural responses toward people (Krueger and Rothbart, 1988; Quinn *et al*., 2009; Mende-Siedlecki, 2018). Parents generally possess ample individuating information about their own children but less for other children (Eccles *et al*., 1990). The personalized information of one’s own children (e.g. knowing their son likes dolls) thus might decrease the likelihood for parents to resort to gender stereotypes (e.g. expecting their son to play with cars) when they think or make judgments about their own children compared to other children (Rubinstein *et al*., 2018). There is some evidence to support this claim. One cross-sectional study among Swedish mothers found that parents of 5-year-olds described their children as less rough than parents of younger children expected for their children at the age of 5 years (Servin and Bohlin, 1999). This finding indicated that parents’ expectations may partly depend on their own children’s preferences and characteristics, despite expecting different behaviors when individuating information about their child at that age was not available. Moreover, individuating information about parents’ own child was found to have a stronger effect on parents’ judgments of their child’s math, sports and social abilities than parents’ gender-stereotyped expectancies (Jacobs and Eccles, 1992).

Relatedly, self-serving bias may cause parents to respond less negative or strong to their own child’s gender nonconformity than to gender nonconformity in unknown children. The self-serving bias entails that people ascribe their successes to internal characteristics and their failures to external factors to maintain a positive self-concept (Bradley, 1978; Zuckerman, 1979). Parents might view their children as an extension of themselves, and therefore, their self-serving bias extends to their child’s behaviors as well (Sedikides *et al*., 1998; Montemayor and Ranganathan, 2012). Accordingly, parents tend to ascribe negative behavior by their own children more to external factors than similar behavior from unknown children, which they often attribute more to internal factors (Larrance and Twentyman, 1983; Montemayor and Ranganathan, 2012). Moreover, internal attributions evoke more negative parenting reactions than external attributions (Bugental and Corpuz, 2019). Therefore, one could assume that parents react more negatively toward unknown children’s gender-nonconforming behaviors than their own children’s nonconforming behaviors, since parents attribute this behavior more to internal characteristics for unknown children.

On the other hand, parents, particularly those parents with strong gender stereotypes, might react more negative to gender-stereotype violations by their own children *vs* other children. It was suggested that parents might fear that they or their child will be stigmatized and bullied when their child exhibits gender-nonconforming behaviors (Rudman and Fairchild, 2004; Goldberg, 2009; Averett, 2016; Abreu *et al*., 2022). In order to prevent this, parents might, for instance, set rules for the display of gender nonconformity by allowing gender-nonconforming behaviors indoors but prohibiting the display of gender nonconformity outside the house (Rahilly, 2015; Scheibling, 2022). Thus, parents might respond stronger to gender-stereotype violations of their own *vs* other children because they want to protect their children from social backlash.

### Neural responses toward the violation of gender stereotypes

One way to measure parents’ reaction to gender-stereotype violations is by using neuroscientific methods to examine parents’ brain processes. Importantly, this method has been found to be a more robust predictor of parents’ gender socialization practices with their children than behavioral measurements of parents’ reactions to gender-stereotype violations or parents’ implicit gender stereotypes (Endendijk *et al*., 2019b). Parents’ neural responses to gender stimuli have most often been examined by comparing patterns of neural activation around the presentation of stimuli, so-called event-related potentials (ERPs). Previous ERP studies have identified several components that are of interest when examining parents’ neural responses toward the violation and confirmation of gender stereotypes. For instance, the P1, N1 and P2 components have been associated with early attentional and information processing and the former two are considered proxies for activity in the dorsal visual stream (Di Russo *et al*., 2003; Novitskiy *et al*., 2011). Regarding the P1, angry female faces (gender-stereotype violations) were found to elicit higher P1 amplitudes than happy female faces (gender-stereotype confirmations) (Liu *et al*., 2017). The N1 has been found to be larger toward expectancy-violating trials than expectancy-confirming trials (Dickter and Gyurovski, 2012; Portengen *et al*., 2022). Results regarding the P2 have been less consistent, with studies finding larger, smaller, and no difference in P2 amplitudes toward the violation *vs* confirmation of social expectations (Jerónimo *et al*., 2017; Yang *et al*., 2020; Portengen *et al*., 2022).

Apart from these three early components, two later neural components have also frequently been implicated in gender stereotype research, namely, the P3 and the late positive potential (LPP). First, the P3 has mostly been associated with attention to unexpected events (Polich, 2007), with higher amplitudes commonly reflecting the level of stimulus-evoked surprise (Mars *et al*., 2008). Moreover, P3 responsivity has been thought to index the updating of a memory representation evoked by the level of expectancy violation of the presented stimulus (Polich, 2007). The P3 has indeed been found to be larger during expectancy-violating trials than expectancy-confirming trials (Bartholow and Dickter, 2007). Lastly, the LPP is an occipital-parietal located component which has been coupled with an extensive brain network of cortical and subcortical structures that differentially respond depending on the valence of a stimulus (Liu *et al*., 2012). The LPP functions as a measure of motivational salience (Hajcak *et al*., 2009) and has been found to be larger toward both gender-stereotype confirmations and violations (Osterhout *et al*., 1997; Liu *et al*., 2017). In sum, several early (P1, N1 and P2) and late (P3 and LPP) components have been identified in earlier research on gender-stereotype violations, which are related to attentional and salience processing of (unexpected) events and the updating of gender-related schemas in the brain.

### The role of gender in the neural processing of stereotype violations

Gender of the parent (i.e. perceiver) as well as gender of the child (i.e. the target) also plays a pivotal role in the neural processing of gender-stereotype violations. In general, gender-stereotyped expectations and gender norms are more restrictive for boys than for girls (Sandnabba and Ahlberg, 1999; Kane, 2006). Moreover, parents are generally more accepting their daughter’s gender-nonconforming behaviors than that of their sons (Solebello and Elliott, 2011). This can also be seen on a neural level: boys with internalizing problem behaviors (e.g. sadness, anxiety; stereotype-violating) elicited larger N1 amplitudes than boys with externalizing problem behavior (e.g. aggression, anger; stereotype-confirming), but this difference was not found for girls (Portengen *et al*., 2022). This indicates that the violation of gender stereotypes might be viewed as more negative for boys than for girls and would therefore evoke stronger neural responses in parents.

Mothers and fathers might also differ in their neural processing of gender stereotypes. In general, fathers have stronger gender-stereotyped expectations of their children (Kollmayer *et al*., 2018), which is also reflected in their stronger neural responses toward (gender) stereotype violations (Proverbio *et al*., 2018). Moreover, men fear being evaluated as gender atypical more than women (Vandello *et al*., 2008) are more likely to reject child nonconforming behaviors and to hold themselves accountable for their son’s nonconforming behaviors (i.e. feeling like a bad father when their son is gender atypical) (Kane, 2006). However, fathers and mothers are equally likely to respond negative toward their children’s gender-nonconforming behaviors (Grossman *et al*., 2005). Thus, based on the majority of studies on this topic, we expect fathers to respond stronger to children’s gender-stereotype violations than mothers.

### Gender cognitions and the neural processing of gender stereotypes

Moreover, individual differences in the strength of gender-stereotyped expectations can play a role in parents’ neural responses toward gender-stereotype violations. Indeed, several studies have found indications that ERP amplitude differences were related to a person’s gender cognitions. For example, a larger P1 amplitude toward gender stereotype-violating than gender stereotype-confirming preschoolers was associated with stronger implicit gender stereotypes (Portengen *et al*., 2022). Moreover, mothers’ P3 activity elicited by gender-stereotype violations and confirmations was found to be related to their level of gender stereotypes (Endendijk *et al*., 2019b). Additionally, differences in LPP amplitudes elicited during stereotype-confirming and stereotype-violating trials have been found to be related to a person’s level of hostile sexism (Canal *et al*., 2015) and gender attitudes (Portengen *et al*., 2022). It is thus important to take parents’ gender cognitions into account when examining parents’ neural responses toward gender-stereotype violations.

### Current study

In sum, there are indications that parents’ gender, children’s gender and parents’ gender cognitions play a role in parents’ neural processing of gender-stereotype violations by children. Moreover, differences in neural responses toward gender-stereotype violations *vs* confirmations in children have been found in the domains of toy preferences (Endendijk *et al*., 2019a, 2019b) and problem behaviors (Portengen *et al*., 2022). However, thus far, most studies have focused on parents’ neural responses toward the violation and confirmation of gender-stereotyped expectations of unknown children instead of parents’ own sons and daughters. Therefore, the primary aim of this study was to examine whether mothers and fathers with a son and a daughter differed in neural responses toward the violation *vs* confirmation of gender stereotypes by their own *vs* unknown children. A secondary aim was to examine whether this difference depended on parents’ own gender, the child’s gender, or parents’ gendered attitudes.

With regard to the primary aim, expectations were that parents would exhibit stronger ERPs in response to gender-stereotype violations than confirmations. In addition, two competing hypotheses were tested with regard to the difference between own and unknown children: (i) parents show stronger brain responses (ERP’s) to gender-stereotype violations of their own children, than to gender-stereotype violations of other children and (ii) parents show less strong brain responses toward gender-stereotype violations of their own children compared to other children. With regard to the secondary aim, we expected that parents’ neural responses toward the violation of gender stereotypes would be stronger for boys than girls (both own and other children). Moreover, we expected fathers to differentiate more on a neural level between gender-stereotype confirmations *vs* violations by both own and other children than mothers. Third, we expected that parents with stronger gender attitudes would show stronger neural responses toward the violation *vs* confirmation of gender stereotypes of both own and other children.

This study examined gender stereotypes in two domains, namely, toy preference and (problem) behaviors. Regarding the former, there is ample evidence that parents and non-parents have strong stereotypical ideas around the appropriateness of toys for boys and girls (e.g. Blakemore and Centers, 2005; Freeman, 2007; Weisgram and Bruun, 2018). For example, parents rate dolls and jewelry as more appropriate for girls, whereas boys are expected to like cars and toy soldiers more than girls (Blakemore and Centers, 2005). The rationale for choosing to include (problem) behaviors was two-fold. First, there are clear gender stereotypes about social-emotional behavior, with boys being expected to show more anger and aggression and girls being allowed to show more internalizing emotions, such as sadness and fear (Brody and Hall, 2008). Moreover, parents have explicit gendered expectations about children’s behavioral traits, for example, expecting girls to be gentle and boys to be noisy (Maccoby and Jacklin, 1974; Martin, 1995). Moreover, the gender-stereotyped expectations associated with these emotions and behaviors have been found to predict later levels of gender-typed problem behaviors in children (Chaplin *et al*., 2005). The gendered emotions and behavioral traits identified in previous research overlap with the problem behaviors that were included in this study. Second, the behaviors words included in this study have been rated as most male-typed (externalizing) and female-typed (internalizing) behaviors by a group of young adults in a previous study (Portengen *et al*., 2022). Moreover, in this same study, the combination of child pictures and behavior words has been found to elicit ERP mean amplitude congruence effects in non-parents.

## Methods

### Participants

Dutch families with a son and a daughter between the ages 3 and 6 years were invited to partake in this study. A total of 74 families were included which consisted of both a mother and a father, with at least one daughter (*M*_age_ = 4.23, s.d._age_ = 1.14) and one son (*M*_age_ = 4.27, s.d._age_  = 1.18) within the target age range and who had sufficient knowledge of the Dutch language to complete the tasks. This within-family design was used to decrease the possibility that differences in the neural processing of gender-stereotype violations by boys and girls are evoked by other factors than child gender (McHale *et al*., 2003; Endendijk *et al*., 2018). Exclusion criteria were a neurological disease (e.g. Parkinson disease and multiple sclerosis) or a history of epileptic seizures. Data collection took place between August 2020 and June 2022. The sample size was a priori determined based on previous electroencephalography (EEG) studies with a similar design that recruited between 25 and 60 participants to detect medium effect sizes (Endendijk *et al*., 2019b; Yang *et al*., 2020). Because this study included between-person (father, mother) comparisons in its statistical models, 74 families (148 participants) were recruited for this study.

### Procedure

Families were recruited through the researchers’ personal networks, via information posters at child daycare centers and primary schools, and by using social media advertisements. Parents could express their interest in participating in the study via e-mail or through an online application form, after which they received an information letter containing detailed information about the procedures and privacy regulations of the study. If parents agreed, an appointment was made for a home visit, during which fathers and mothers sequentially underwent EEG examination while performing an impression formation task (IFT). The parent who was not completing the EEG part of the study was participating in an observation study with their son and daughter. Parent gender and EEG task block order were counterbalanced, so that, for instance, in half of the families, the mother commenced with the EEG examination, and half of these mothers started with the toy preference block, whereas the other half started with the behavior block. Prior to the home visit, parents were asked to upload a picture of their participating son and daughter with a neutral facial expression onto a secured platform (see Supplementary Materials for more information about the instructions provided for parents). These pictures were included in the IFT (see the Instruments section). Written informed consent was obtained from both parents pretesting. Parents also gave consent for their participating children. Families received a gift card worth 25 euros for participation, and the children received a small gift. Ethical approval was granted by the faculty ethics review board from the Social and Behavioral Sciences faculty at Utrecht University (19–232). This study was not preregistered.

### Instruments

#### IFT

The IFT used in this study was based on a previously validated IFT task for assessing ERP responses toward gender stereotypes (Portengen *et al*., 2022). Parents passively viewed 20 pictures of Caucasian children with a neutral facial expression (10 boys and 10 girls) and the pictures of their children (one son and one daughter), which were combined with a word stimulus, and were told to quickly form an impression about the child based on the provided information. The 20 unknown children were selected from the Child Affective Facial Expression database (LoBue and Thrasher, 2015) based on who were the most clearly male-typed boys and female-typed girls (Endendijk *et al*., 2019a). Brightness levels were altered for both the pictures of unknown children and the pictures of parents’ own children, so that their mean luminance was between 190 and 205.

**Fig. 1.**
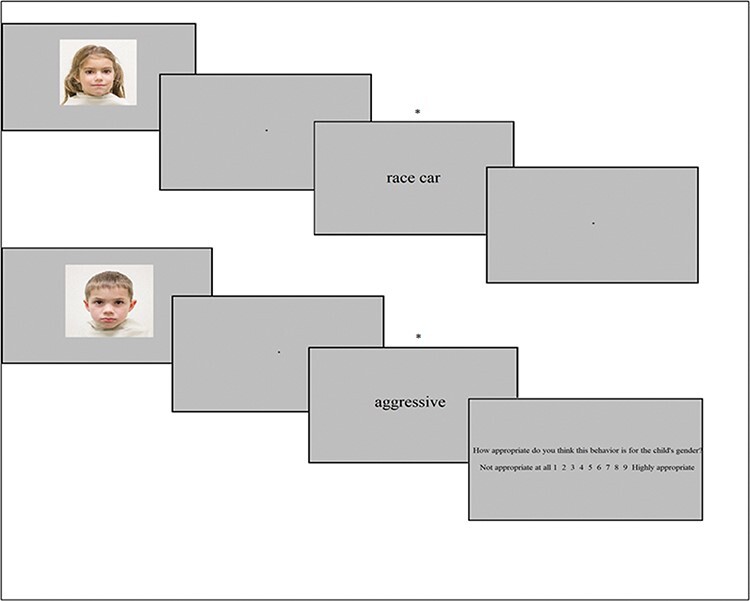
Visual example of IFT with the upper representing an incongruent trial without question and the lower representing a congruent trial with appropriateness question. *Note*. The asterisk in the 1 indicates the timing of the trigger signals around which the EEG segmentations are constructed.

After each picture, a toy (block 1) or behavior (block 2) word appeared, respectively describing a toy the child loves to play with or problem behavior that the child frequently exhibits (Figure 1). The toy word stimuli set contained 10 masculine toy words (crane, tractor, race car, garage, toolkit, soccer, digger, fire truck, pirates costume and helicopter) and 10 feminine toy words (tea set, princess dress, hula-hoop, doll clothes, barbie, play kitchen, jewelry and doll house) that were indicated as clearly masculine and feminine toys in prior studies (Blakemore and Centers, 2005; Endendijk *et al*., 2014, 2019a). The behavior word set contained 20 words derived from the internalizing and externalizing behavior scales from the Child Behavior Checklist (Achenbach, 1999). In this set, 10 externalizing behavior words were selected as describing male-typed behavior (violent, fighting, threatening, kicking, agitated, inattentive, noisy, cruel, disobedient and aggressive) and 10 internalizing behavior words were selected as describing female-typed behavior (dependent, shy, unhappy, depressed, sad, fearful, worried, ashamed, avoidant and sensitive). These behavior words were previously rated as most male-typed and female-typed externalizing and internalizing behaviors, respectively (Portengen *et al*., 2022).

Each block consisted of 240 trials, 120 trials with unknown children and 120 trials with parents’ own children. Each unknown child picture appeared a total of 12 times, 6 times with a word that was congruent with gender stereotypes (e.g. a boy face paired with “race car,” a girl face paired with “shy”) and 6 times with a word that was incongruent with gender stereotypes (e.g. a boy face paired with “princess doll,” a girl face paired with “disobedient”). The words were pseudo-randomly assigned to the unknown child pictures, ensuring that no word stimulus appeared twice with the same child face picture. Parents’ own child pictures appeared 60 times per block, 30 times paired with congruent words and 30 times paired with incongruent words. Before each trial, a fixation cross was presented for a varying amount of time (800, 900, 1000, 1100 or 1200 ms, randomly chosen), after which a face picture (1000 ms, width:13.3 cm, height: 9.2 cm) appeared, superimposed in a gray background (191;191;191). A jittered interstimulus interval was assigned for 200, 225, 250, 275 or 300 ms, after which the word stimulus was presented in black for 1000 ms (Cambria, font size 55). The word stimuli were trigger coded so that ERPs were segmented around the presentation of the word stimulus.

In half of the trials, a question appeared after the face–word combination (in Cambria font size 24). This question is as follows: “How appropriate do you think this toy/behavior is for the child’s gender?” This question was added to retain participants’ attention and as a measure of parents’ explicit gender attitudes about the appropriateness of the displayed toy and problem behavior words. Participants were instructed to rate the appropriateness of each combination on a scale from 1 (not appropriate at all) to 9 (highly appropriate) by pressing the numbers on the keyboard. The question was presented until the participant pressed a response key (1–9). The task was designed in E-Prime v3.0 (Taylor and Marsh, 2017) and presented electronically on a 14-inch laptop. The task took approximately 40 min to complete, depending on how quickly a parent responded to the posed questions and the length of their self-paced break within each block and in between the two blocks (3 breaks in total).

#### Parents’ gender attitudes about children’s toy preference and behavior

Appropriateness ratings of the IFT face–word combinations were extracted and used as a measure of parents’ level of gender attitudes about toy preference and problem behaviors. Gender attitudes about toy preference were calculated by subtracting the average appropriateness scores on incongruent trials during the toy block from the average appropriateness scores on congruent trials during the toy block (Portengen *et al*., 2022). The same scores were calculated for parents’ gender attitudes about problem behavior. A higher positive score meant that parents rated stereotype-congruent child–toy or child–behavior combinations as more appropriate than stereotype-incongruent child–toy or child–behavior combinations.

### Electroencephalographic measurement and preprocessing

BioSemi ActiveTwo Ag-AgCl pin electrodes and hardware was used to record EEG from 32 scalp sites (BioSemi, 2011). The electrodes were positioned using the 10- to 20-electrode system using a nylon electrode cap (Klem *et al*., 1999). EEG signals were sampled at 2048 Hz, amplified and bandpass filtered at DC-400 Hz. Eye movements were recorded by placing four sintered Ag-AgCl electrodes above and below the left eye and just outside the outer corner of each eye.

**Fig. 2.**
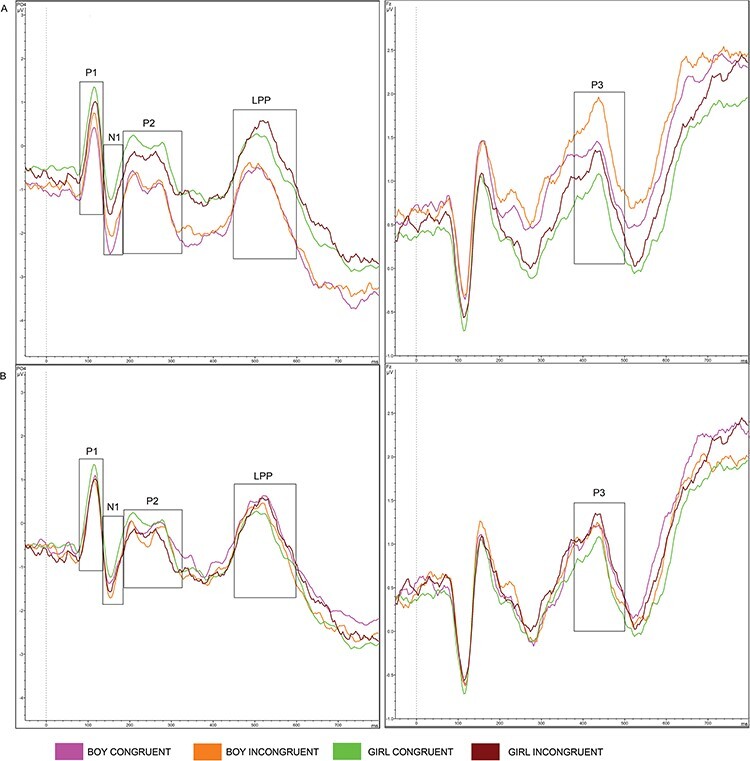
Total average waveforms during the toy block, separate per child type: (A) unknown and (B) own.

**Fig. 3.**
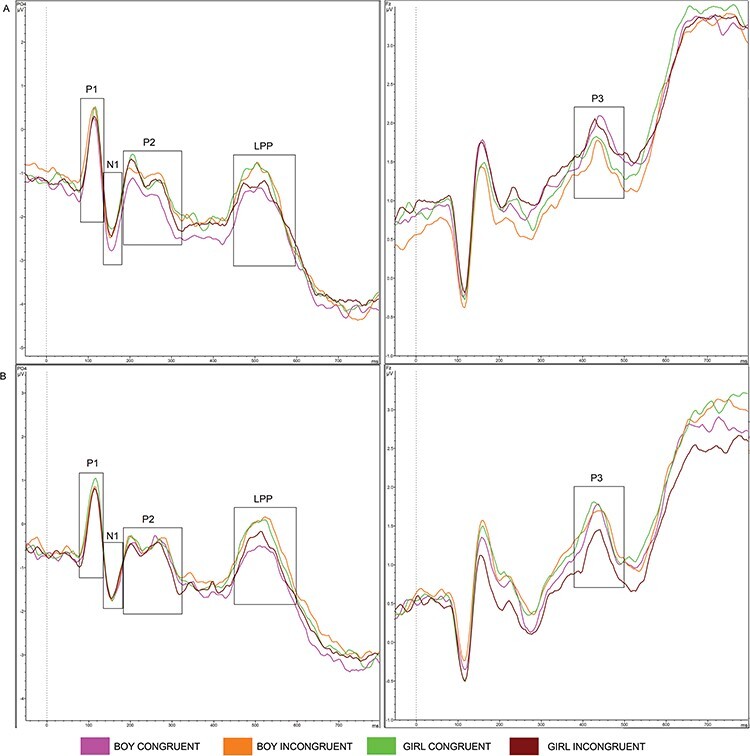
Total average waveforms during the behavior block, separate per child type: (A) unknown and (B) own.

EEG data were offline downsampled to a sampling rate of 256 Hz, followed by bandpass filtering of 0.1–30 Hz. Each parent’s data were re-referenced to the average activity in all channels. Data were cut into segments of −200 to 1000 ms, time-locked to the onset of the word stimuli. A baseline correction of −200 to 0 ms was applied to correct for baseline differences in voltage and drift between trials and electrodes. The Gratton and Coles method was applied to correct for ocular artifacts (Gratton *et al*., 1983). Artifacts rejection was done semiautomatically, which meant that trials were marked as bad if the voltage step exceeded 50 µV/ms, with a maximum allowed difference of 1000 µV in intervals within a 200-ms window or with activity in intervals below 0.5 µV. Bad trials were visually inspected and discarded if the artifact was present across two or more electrodes or in one of the electrodes of interest. A channel was discarded from preprocessing and further analysis for that participant if artifacts were presented in more than 25% of the trials and if this was not one of the electrodes of interest. Participants were excluded when they had significant noise in one of the electrodes of interest or when there were less than 10 valid trials included in the average segments. The remaining data for each participant were averaged into one grand average waveform per condition. Total average waveforms were created from each participant’s grand average waveform. These waveforms were visually inspected to select time windows and electrodes for the ERP components of interest. The electrodes and time windows with the largest amplitudes were selected; see Table 1 for an overview of the time windows and electrodes per condition and Figures 2 and 3 for the total average waveforms, separate for each block (toy and behavior). Figure S2 depicts the total average waveforms in all electrodes of interest.

**Table 1.** Overview of the time windows and electrodes for the ERPs of interest

ERPs	Time window (ms)	Electrodes	Similar to ERPs measured in
P1	80–135	Pz, P3, P4, PO3, PO4, O1, Oz and O2	He *et al*. (2009)
N1	135–180	Pz, P3, P4, PO3, PO4, O1, Oz and O2	Portengen *et al*. (2022)
P2	185–325	Pz, P3, P4, PO3 and PO4	Williams and Themanson (2011)
P3	380–500	FC1, FC2 Fz and Cz	Endendijk *et al*. (2019b)
LPP	450–600	P3, P4, PO3 and PO4	Breton *et al*. (2019) and Ito *et al*. (2004)

### Data analysis

Mean amplitudes per subject and condition were exported from Brainvision Analyzer and imported into R v. 4.2.1 (R Core Team, 2013). Multilevel models were fit by maximum likelihood using the *lme4* package (Bates *et al*., 2018). Separate models were run per ERP (P1, N1, P2, P3 and LPP) and block (toy and behavior). In these models, ERP mean amplitude was the outcome variable. Since we used fathers and mothers from the same family, parents were nested within families. Since each ERP was measured with multiple electrodes, electrode was nested within the parent. Congruence (congruent/incongruent), child gender (boy/girl) and child type (own/unknown) were added as factors of interest. Parent gender (mother/father) and parents’ gender attitudes were added as independent variables if they significantly improved model fit. Moreover, since we were primarily interested in congruence effects, interaction terms were added for congruence and child gender, child type, parent gender, and gender attitudes if these would significantly improve the model fit. Model fit improvement was determined by a significant decrease in the Akaike information criterion and Bayesian information criterion (Matuschek *et al*., 2017). Model fits were compared with the anova function. Finally, to control for type 1 error rates, the highest order interaction was additionally added as a random slope per participant, as recommended by Volpert-Esmond *et al*. (2021). Degrees of freedom and *P*-values of fixed effects were estimated using Satterthwaite’s method. Residuals and histograms were visually inspected for homoscedasticity and normality of residuals and checked for residual outliers.

## Results

### Data inspection and descriptive statistics

From the initial 148 parents who participated in this study, nine parents (six mothers) were excluded from further analyses, since no or insufficient EEG data were collected (*n* = 5) or because of noisy data (*n* = 4). For four participants (two mothers), the appropriateness ratings were not saved due to technical problems. This led to the inclusion of 135 parents in the main analyses. Table 2 contains an overview of mothers’ and fathers’ descriptive characteristics. Fathers were on average older than mothers (*P =* 0.018). Mothers and fathers achieved similar educational levels but differed in their number of paid working hours, with mothers working significantly less hours than fathers (*P < *0.001).

**Table 2.** Participant information separate for mothers and fathers

	Mothers (*n* = 65)	Fathers (*n* = 70)	Test statistics	*P*-value
Age, *M* (s.d.) [range]	35.83 (3.53) [29–45]	37.54 (4.76) [30–53]	*t*(127) = 2.39	0.019
Educational attainment, *n* (%)			*χ^2^*(3) = 3.53	0.317
High school	2 (3.1)	2 (2.9)		
Secondary vocational education	12 (18.5)	20 (28.6)		
Bachelor’s degree	18 (27.7)	23 (32.9)		
Master’s degree	33 (50.8)	25 (35.7)		
Paid working hours, *n* (%)			*χ^2^*(5) = 61.57	<0.001
No paid working hours	5 (7.7)	0 (0.0)		
1–10	1 (1.5)	0 (0.0)		
11–20	12 (18.5)	1 (1.4)		
21–30	29 (44.6)	4 (5.7)		
31–40	17 (26.2)	55 (78.8)		
40+	1 (1.5)	10 (14.3)		
GAT, *M* (s.d.) [range]	3.10 (1.56) [−0.06–6.65]	3.13 (1.22) [−0.15–5.38]	*t*(121) = −0.04	0.967
GAB, *M* (s.d.) [range]	0.72 (0.73) [−0.83–2.47]	0.73 (0.73) [−0.82–3.00]	*t*(132) = 0.11	0.915

Abbreviations: GAT, gender attitudes about toys; GAB, gender attitudes about child problem behavior.

Parents rated toy–child combinations that confirmed gender stereotypes (*M* = 7.42, s.d. = 0.72) as more appropriate than toy–child combinations that violated gender stereotypes (*M* = 4.54, s.d. = 1.31; *t*(134) = 24.07, *P < *0.001). Similarly, parents rated child–behavior combinations that confirmed gender stereotypes (*M* = 4.23, s.d. = 1.12) as more appropriate than child–behavior combinations that violated gender stereotypes (*M* = 3.50, s.d. = 1.08; *t*(134) = 11.58, *P < *0.001). Parents had significantly stronger gender attitudes about toys paired with unknown children (*M* = 3.17, s.d. = 1.47) compared to their own children [*M* = 2.73, s.d. = 1.50; *t*(134) = 3.23, *P =* 0.002]. Similarly, parents held stronger gender attitudes about problem behaviors paired with unknown children (*M* = 1.14, s.d. = 0.77) compared to their own children [*M* = 0.30, s.d. = 1.15; *t*(134) = 7.40, *P < *0.001].

All model residuals revealed no violation of the assumptions of homoscedasticity and normality of residuals. Further examination of the *Q*–*Q* plot of standardized residuals revealed one outlier (>6 or < −6) in the P1 and N1 model for the toy block and the P1 and P2 models for the behavior block. Excluding the outliers only changed the slope, but not the significance of findings reported in the result section.

### Parents’ neural responses toward the violation of gender stereotypes about toys

Table S1 contains all uncorrected statistics for the five components during the toy trials. No significant congruence main effects, nor interactions with congruence, were found for the N1, P2, P3 or LPP components during the toy block (see the Supplementary Materials for a more detailed description of findings during the toy block).

#### P1

Significant main effects were found for congruence [*β* = 0.05, *t*(135) = 2.26, *P =* 0.025], child gender (*β* = 0.04, *t*(135) = 2.01, *P =* 0.046) and child type [*β* = 0.08, *t*(135) = 3.09, *P =* 0.002]. P1 mean amplitudes were stronger during incongruent trials than congruent trials, during trials that included girls than boys and during trials that included parents’ own *vs* unknown children. Moreover, a significant interaction was found between congruence and child gender [*β* = −0.06, *t*(135) =  −2.49, *P =* 0.014]. Decomposing the interaction effect revealed that for trials with girls, parents’ P1 mean amplitudes were larger during congruent than incongruent trials [*β *= −0.03, *t*(135) =  −2.03, *P =* 0.044], but this effect was not significant for trials with boys [*β* = 0.02, *t*(135) = 1.65, *P =* 0.101].

### Parents’ neural responses toward the violation of gender stereotypes about problem behavior

Since there were indications in the N1, P2 and LPP mean amplitudes that congruence effects had carried over from the preceding components, we included the preceding component as a control variable in the models. The uncorrected results can be found in the Supplementary Materials and Table S2. Table 3 contains all statistics for the five components during the behavior trials, with N1, P2, and LPP results corrected for the preceding component. Only the congruence effects are listed below as these provided tests of our hypotheses, other findings can be found in the Supplementary Materials. No effects of congruence were found for N1 and P2.

**Table 3.** Results from the multilevel models examining congruence effects on ERP mean amplitudes during behavior trials corrected for the preceding components

P1	*b*	$\boldsymbol{\beta}$	SE	*t*(d*f*)	*P*
Congruence	0.41*	0.07	0.14	2.81 (135)	0.006
Child gender	0.07	0.01	0.10	0.69 (135)	0.494
Child type	0.57*	0.09	0.16	3.60 (135)	<0.001
Parent gender	0.23	0.04	0.29	0.80 (77)	0.428
GAB	−0.06	−0.02	0.21	−0.30 (135)	0.763
Congruence × GAB	−0.14*	−0.03	0.07	−1.98 (135)	0.050
Congruence × child gender	−0.40*	−0.06	0.15	−2.68 (135)	0.008
Congruence × child type	−0.33	−0.05	0.17	−1.88 (135)	0.062
Congruence × parent gender	−0.02	−0.003	0.10	−0.24 (135)	0.813
Child gender × child type	−0.01	−0.002	0.16	−0.09 (135)	0.930
Congruence × child gender × child type	0.24	0.03	0.26	0.91 (135)	0.367
**N1** ^**a**^	***b***	$\boldsymbol{\beta}$	**SE**	***t*(d*f*)**	***P***
Congruence	0.11	0.02	0.09	1.24 (135)	0.217
Child gender	0.06	0.01	0.06	1.08 (135)	0.283
Child type	0.14*	0.03	0.07	2.45 (135)	0.016
Parent gender	−0.30	−0.05	0.19	−1.59 (67)	0.116
GAB	−0.32*	−0.07	0.14	−2.37 (135)	.020
Congruence × GAB	−0.05	−0.01	0.05	−0.90 (135)	0.372
Congruence × child gender	−0.02	−0.003	0.09	−0.26 (135)	0.799
Congruence × child type	0.07	0.01	0.09	0.82 (135)	0.413
Congruence × parent gender	−0.11	−0.01	0.08	−1.43 (135)	0.154
**P2^a^**	***b***	$\boldsymbol{\beta}$	**SE**	***t*(df)**	***P***
Congruence	0.04	0.01	0.09	0.39 (135)	0.700
Child gender	−0.06	−0.01	0.07	−0.84 (135)	0.403
Child type	−0.02	−0.004	0.10	−0.19 (135)	0.849
Parent gender	0.13	0.02	0.13	0.94 (67)	0.350
GAB	−0.09	−.02	0.10	−0.77 (135)	0.440
Congruence × GAB	0.02	−0.004	0.08	0.21 (135)	0.834
Congruence × child gender	−0.05	−0.01	0.11	−0.46 (135)	0.647
Congruence × child type	0.03	0.004	0.13	0.19 (135)	0.849
Child gender × child type	−0.04	−0.002	0.10	−0.69 (135)	0.873
Child type × GAB	0.26*	0.06	0.09	2.71 (135)	0.008
Congruence × child gender × child type	0.02	0.004	0.16	0.23 (135)	0.822
Congruence × child type × GAB	−0.09	−0.02	0.12	−0.75 (135)	0.455
**P3**	***b***	$\boldsymbol{\beta}$	**SE**	***t*(df)**	***P***
Congruence	−0.13	−0.03	0.12	1.14 (135)	0.256
Child gender	−0.11	−0.03	0.12	−0.98 (135)	0.328
Child type	−0.24*	−0.06	0.11	−2.25 (135)	0.026
Congruence × child gender	0.25	0.05	0.14	1.84 (135)	0.068
Congruence × child type	0.33*	0.07	0.15	2.25 (135)	0.026
Child gender × child type	0.19	0.04	0.20	0.93 (135)	0.352
Congruence × child gender × child type	−0.66*	−0.10	0.30	−2.19 (135)	0.031
**LPP^a^**	***b***	$\boldsymbol{\beta}$	**SE**	***t*(df)**	***P***
Congruence	−0.05	−0.01	0.09	−0.43 (135)	0.590
Child gender	−0.04	−0.01	0.11	−0.36 (135)	0.720
Child type	0.01	0.001	0.13	0.08 (135)	0.938
Congruence × child gender	0.05	0.01	0.13	0.40 (135)	0.693
Congruence × child type	0.34*	0.04	0.15	2.26 (135)	0.026
Child gender × child type	0.22	0.03	0.17	1.31 (135)	0.194
Congruence × child gender × child type	−0.37	−0.03	0.20	−1.82 (135)	0.071

*Note*: Child type refers to the difference between parents’ own children and unknown children. Congruent, boy, unknown child and father were the reference categories for congruence, child gender, child type, and parent gender, respectively.

a Model outcomes are corrected for the preceding component.

* Significant effects with *P < *0.05.

Abbreviation: GAB, gender attitudes about child problem behavior; SE, standard error.

#### P1

A main effect of congruence [*β* = 0.07, *t*(135) = 2.81, *P =* 0.006] and child type [*β* = 0.10, *t*(135) = 3.60, *P < *0.001] was found on P1 mean amplitudes. P1 amplitudes were stronger during incongruent trials than during congruent trials and during trials that contained parents’ own children compared to trials with unknown children. Moreover, two variables significantly interacted with congruence, namely child gender [*β* = −0.06, *t*(135) = −2.68, *P =* 0.008] and parents’ gender attitudes about child problem behavior [*β* = −0.03, *t*(135) = −1.98, *P =* 0.050].

Decomposing the first interaction effect revealed a significant main effect of congruence on boy trials [*β* = 0.06, *t*(174) = 2.22, *P =* 0.028]. P1 amplitudes were stronger during trials in which boys were paired with internalizing behavior words (incongruent trials) than when boys were paired with externalizing behavior words (congruent trials). This main effect of congruence was not significant for girls [*β* < −0.01, *t*(178) = −0.03, *P =* 0.974].

**Fig. 4.**
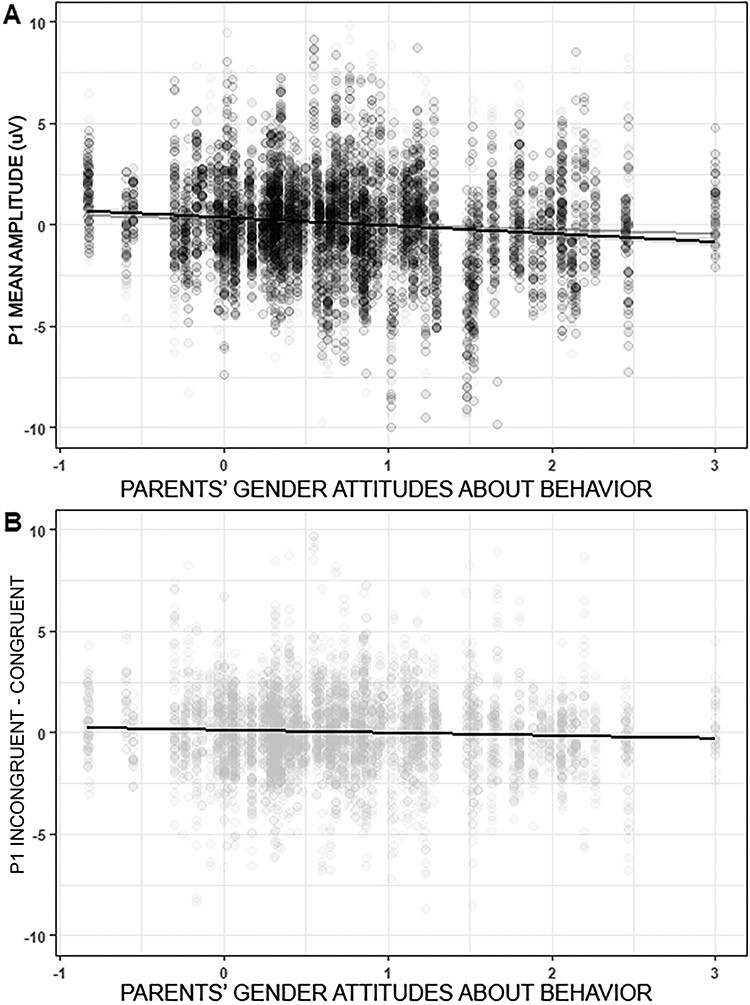
Effect of parents’ gender attitudes about child problem behavior on parents’ P1 amplitudes during congruent and incongruent trials. (A) Separate effects of congruent (black) and incongruent (gray) trials. (B) Difference in P1 amplitudes during incongruent–congruent trials.

With regard to the interaction between gender attitudes and congruence, mean amplitudes during incongruent trials were subtracted from mean amplitudes during congruent trials (see Figure 4). For parents with stronger gender attitudes about problem behaviors (i.e. rated incongruent behavior as less appropriate for a child’s gender than congruent behaviors), their P1 amplitudes toward incongruent trials were larger than toward congruent trials, whereas parents with less strong gender attitudes had larger P1 amplitudes toward congruent than incongruent trials.

#### P3

The multilevel analysis with P3 mean amplitudes as a dependent variable revealed a main effect of child type [*β* = −0.06, *t*(135) = −2.25, *P =* 0.026]. P3 mean amplitudes were larger during trials that included unknown children than their own children, regardless of which behavior they were paired with. Moreover, a significant two-way interaction was found for congruence with child type [*β* = 0.07, *t*(135) = 2.25, *P =* 0.026]. This interaction was subsumed under a significant three-way interaction between congruence, child gender, and child type [*β* = −0.10, *t*(135) = 2.19, *P =* 0.031].

Analyses were run separate for own and unknown children. The model with P3 mean amplitudes during trials with parents’ own children revealed no main effect of congruence [*β* = 0.05, *t*(135) = 1.67, *P =* 0.097] nor was the interaction between congruence and child gender significant [*β* = −0.09, *t*(135) = −1.68, *P =* 0.095]. For unknown children, however, a significant interaction between congruence and child gender was found [*β* = 0.05, *t*(135) = 1.99, *P =* 0.048]. Subsequent analyses revealed that parents had larger P3 amplitudes toward incongruent trials than congruent trials for girls that were not their own children [*β* = 0.12, *t*(135) = 0.92, *P =* 0.359], but the reverse was found for boys [i.e. larger P3 amplitudes toward congruent than incongruent trials; *β* = −0.013, *t*(135) = −1.14, *P =* 0.256], although neither association reached statistical significance. In other words, externalizing behavior words elicited larger P3 mean amplitudes than internalizing behavior words when combined with unknown children.

#### LPP

A significant interaction emerged between congruence and child type [*β* = 0.04, *t*(135) = 2.26, *P =* 0.025]. Decomposing the interaction effect revealed a main effect of congruence in LPP amplitudes during trials that included parents’ own children [*β* = 0.04, *t*(135) = 2.42, *P =* 0.017], but this main effect was not found for trials that included unknown children (*P =* 0.706). Parents’ LPP mean amplitudes were larger during incongruent trials than congruent trials with their own children. Moreover, parents’ LPP responses during incongruent trials were larger toward their own children than unknown children [*β* = 0.05, *t*(137) = 3.90, *P < *0.001], but this difference was not found for congruent trials (*P =* 0.896).

### Sensitivity analysis

To examine whether parents’ ERP responses to gendered behavior were representing parents’ neural processing of children’s actual behavior patterns, zero-order correlations were calculated between the significant ERP mean amplitudes (per condition and per electrode) and parents’ reports of their sons’ and daughters’ internalizing and externalizing behaviors (see Table S3 in the Supplementary Materials). Internalizing and externalizing behavior problems were measured with the 12-item Brief Problem Checklist (Chorpita *et al*., 2010). Correlations between these variables were low (ranging between −0.187 and 0.209), leading us to assume that other factors than their children’s actual behaviors played a role in parents’ ERP responses.

Through close inspection of Figures 2 and 3, differences in baseline amplitudes can be observed, indicating that the baseline correction had not been effective enough to cancel out possible priming effects of the child pictures that preceded the word stimuli. We therefore included the baseline mean amplitudes in the models that were not corrected for the preceding ERP component (i.e. the P1 and P3 models) as recommended by Alday (2019), as an alternative for baseline correction. For all electrodes of interest, baseline mean amplitudes (from −200 ms to 0 ms) were extracted from Brainvision Analyzer and imported in R.

#### Toy stimuli

Including the mean activity at baseline (−200 to 0 ms) as a predictor in the P1 model for the toy block led to non-significant main effects of congruence (*P =* 0.089) and child gender (*P =* 0.116). Moreover, the interaction between congruence and child gender was no longer significant when baseline activity was included as a predictor (*P =* 0.109). The main effect of child type remained significant (*β* = 02, *t*(135) = 5.74, *P < *0.001). With regard to P3 mean amplitudes, when baseline activity was taken into account, P3 mean amplitudes were significantly larger during trials that included unknown children compared to parents’ own children [*β* = −0.06, *t*(135) = −2.33, *P =* 0.021]. No other significant effect emerged.

#### Behavior stimuli

Including the mean baseline activity as a predictor in the P1 mean amplitude model led to a non-significant main effect of congruence (*P =* 0.212). Moreover, the interaction between congruence and gender attitudes (*P =* 0.487) and congruence and child gender (*P =* 0.217) were no longer significant. The main effect of child type remained significant [*β* = 0.04, *t*(135) = 2.10, *P =* 0.038]. With regard to the P3 mean amplitude model, including the baseline mean activity only changed the slope, but not the significance of the findings [main effect child type: *β* = −0.08, *t*(135) = −2.96, *P =* 0.004; interaction congruence, child gender, and child type: *β* = −0.09, *t*(135) = −2.00, *P =* 0.048].

## Discussion

This study examined whether mothers and fathers showed different neural responses toward gender-stereotype violations and confirmations by their own *vs* unknown children. Indeed, LPP responses were larger when parents’ own children violated gender stereotypes about problem behavior than when parents’ own children confirmed these gender stereotypes. Moreover, P1 mean amplitudes were larger toward girls that confirmed gender stereotypes about toys compared to girls who violated gender stereotypes about toys, regardless of whether it concerned parents’ own or other children. Conversely, P1 amplitudes were stronger toward boys who violated gender-stereotyped expectations about problem behaviors than boys who confirmed gender expectations. Additionally, parents with stronger gender attitudes about problem behavior had stronger P1 responses toward children’s behaviors that violated gender stereotypes compared to behaviors that confirmed gender stereotypes. Importantly, all P1 amplitude interaction effects disappeared when controlling for mean baseline activity. Last, although simple main effects were not significant, the significant interaction between congruence, child type, and child gender suggested that P3 amplitudes were stronger toward unknown girls paired with externalizing behavior words than unknown girls paired with internalizing behavior words, but stronger toward unknown boys paired with externalizing behavior words than unknown boys paired with internalizing behavior words. Contrary to expectations, no differences were found between mothers’ and fathers’ neural processing of gender stereotypes.

The current study provided some evidence that parents’ neural responses toward gender-stereotype violations were different for their own children compared to unknown children. Stronger LPP mean amplitudes for behaviors that violated gender stereotypes (compared to stereotype confirmations) were found specifically for parents’ own children. LPP responses reflect salience processing and controlled attentional orienting (Brown *et al*., 2012; Liu *et al*., 2012) and have also been found to be modulated by the salience of gender stereotypes (Rodríguez-Gómez *et al*., 2020). Increased LPP mean amplitudes might thus reflect more top-down processing of gender stereotype-violating problem behaviors performed by parents’ own children. Parents might respond more strongly toward gender-stereotype violations by their own children because they want to protect them from social exclusion and backlash (Skewes *et al*., 2018; Sullivan *et al*., 2018). An important next step is to examine whether LPP responses toward gender-stereotype violations by parents’ own children also relate to gender socialization practices with their children. Regarding this, it has been found that parents’ neural responses to gendered stimuli of other children are associated with parents’ gendered communication with their own children (Endendijk *et al*., 2019b).

There were also some indications that own and unknown children evoked different P3 responses; specifically, externalizing behavior words paired with unknown children evoked larger P3 amplitudes than internalizing behavior words. P3 amplitudes have previously been associated with negatively valanced stimuli (Duval *et al*., 2013; Gyurovski *et al*., 2018). This may indicate that parents evaluate externalizing behavior as more negative than internalizing behavior when these words were paired with other children.

Notably, we only found differences between own and unknown children on the P3 and LPP components, but not on earlier components. Moreover, the effects we did find for own *vs* unknown children were small. This could indicate that familial kinship does not play a large role in the neural processing of gender-stereotype violations. Relatedly, parents evaluated unknown children’s gender-stereotype violations more negatively than their own children’s gender-stereotype violations. Conversely, these findings could indicate that multiple underlying processes, such as individuating information, self-serving bias, or fear of backlash, might simultaneously play a role in parents’ processing of gender-stereotype violations by their own children. These factors might cancel out each other’s reinforcing or attenuating effects on the neural processing of gender-stereotype violations of own *vs* unknown children. Future research could aim to disentangle the effects of these underlying processes (individuating information, self-serving bias, or fear of social backlash) on parents’ reactions to stereotype violations by their own children.

With regard to the role of gender of the children, contrasting evidence was found for the hypothesis that gender stereotype-violating boys evoked stronger neural responses than gender stereotype-confirming boys. Importantly, since the P1 effects disappeared when correcting for baseline (priming) activity, these effects should be interpreted cautiously. In contrast to our hypothesis, parents’ P1 mean amplitudes were larger during gender stereotype-confirming girl-toy combinations (e.g. girl and doll) than gender stereotype-violating girl-toy combinations (e.g. girl and crane). Recent research shows that in early childhood, apart from gender-typed play, both girls and boys develop an increasing interest in and preference for masculine toys (Todd *et al*., 2018). Perhaps parents may find play with gender-atypical toys more important, appropriate or expected for girls than boys. In line with our hypothesis, P1 mean amplitudes were stronger toward gender stereotype-violating boy-behavior combinations (e.g. boy and depression) than gender stereotype-confirming boy-behavior combinations (e.g. boy and aggression). This is in line with previous research indicating that gender norms are more restrictive for boys than girls (Sandnabba and Ahlberg, 1999; Sullivan *et al*., 2018). For boys, there is a greater need to avoid feminine behaviors (Vandello and Bosson, 2013). This fits the idea of precarious manhood, which dictates that manhood is something that is earned through the social display of masculine behaviors, whereas womanhood is something biological and does not need to be reinforced (Vandello *et al*., 2008). In addition, gender-atypical behavior displayed by boys is often heavily penalized and evokes stronger behaviors in adults than gender-atypical display of behaviors by girls (Kane, 2006; Scheibling, 2022).

Some evidence was also found for the hypothesis that parents’ gender cognitions played a role in the neural processing of gender-stereotype violations, specifically in P1 mean amplitudes toward congruent and incongruent child–behavior combinations. Parents with stronger gender attitudes about behavior had larger P1 responses toward gender-stereotype violations compared to gender-stereotype confirmations. This interaction between gender attitudes and P1 amplitudes is similar in direction as the study from Portengen *et al*. (2022), in which adults’ P1 amplitudes were modulated by their level of implicit gender stereotypes. However, in the Portengen *et al*. (2022) study the interaction was found specifically for adults’ implicit gender stereotypes and not for gender attitudes. One reason for the differences between these studies is that in the current study, parents’ implicit gender stereotypes were not assessed and could not be controlled for. Implicit gender stereotypes and gender attitudes are related constructs (Greenwald *et al*., 2002; Gawronski and Creighton, 2013). Therefore, part of the relation found between gender attitudes and P1 mean amplitudes in this study could be explained by parents’ levels of implicit gender stereotypes. Another, reason for the differences between these studies is that the Portengen *et al*. (2022) study focused on non-parents, whereas the current study focused on parents. It might be that for parents the attitudes they have about the appropriateness of certain behaviors for boys and girls plays a more important role in the neural processing of gender-stereotype violations than for non-parents.

Mothers and fathers did not differ in their neural responses toward gender-stereotype violations and confirmations. This contrasts previous literature that found that men and fathers have stronger stereotyped expectations than women and mothers (Endendijk *et al*., 2013; Proverbio *et al*., 2018). Interestingly, fathers and mothers also did not differ in their levels of explicit gender attitudes in this study (i.e. how (in)appropriate they rated gender-stereotype violations), which may explain why no differences were found in their neural responses to gender-stereotype violations as well. It confirms other work that found that fathers and mothers also appear to be equally likely to respond negative toward their children’s gender-nonconforming behaviors (Grossman *et al*., 2005).

It is also important to note that less differences were found in parents’ neural processing of gender-stereotype violations and confirmations regarding children’s toy preferences compared to problem behavior. This partly replicates previous studies that also did not find any effects of gender-stereotype violations regarding toy preferences on ERP mean amplitudes (Endendijk *et al*., 2019b; Portengen *et al*., 2022). The limited effects in the toy domain could be due to a selection bias in participating families. Although the gendered nature of this study was not advertised explicitly, parents could have easily derived that the study revolved around gender from the information available. Therefore, the participating parents may have been more familiar with and interested in gender-neutral parenting. Several families indeed indicated after the study that they tried to raise their children as gender-neutral as they deemed possible. Gender-neutral parenting often revolves around creating a gender-neutral environment for children (e.g. toy availability, room decorations) (Martin, 2005) and places less emphasis on the development of gendered behaviors. Another explanation for the lack of findings in the toy domain could be the different valences of the word categories used in this study. The behavior words have a stronger negative component and might thus elicit stronger reactions in parents. In general, bad things impact people more strongly than good things (Chen and Bargh, 1999; Baumeister *et al*., 2001). The words used in the toy block generally have a positive or neutral connotation and might thus be considered less arousing for parents. The negative valence of stimuli is known to elicit more attention allocation as demonstrated by stronger P1 amplitudes (Smith *et al*., 2003).

Finally, the current study did not find differences in parents’ N1 and P2 mean amplitudes toward gender-stereotype violations and confirmations. With regard to the lack of findings in the N1 domain, some previous studies have found larger mean amplitude toward (gender-) stereotype violations than confirmations (Dickter and Gyurovski, 2012; Portengen *et al*., 2022). Nonetheless, there are also several studies that report no N1 modulation in response to expectancy violations and confirmations (e.g. Leuthold *et al*., 2011; Healy *et al*., 2015). In the current study, N1 mean amplitudes were corrected for the preceding difference in P1 mean amplitudes. When left uncorrected for the previous peak, N1 amplitudes were modulated by interactions between gender attitudes and congruence (stronger N1 differences when parents had more traditional gender attitudes about behavior) and child gender and congruence (congruence effect found for boys but not girls, with larger amplitudes during congruent than incongruent trials). It is possible that some of the N1 effects observed in previous studies may have been carryover effects from P1 mean amplitude differences. The lack of findings in P2 mean amplitude modulations is not surprising considering the inconsistent findings from previous research (Jerónimo *et al*., 2017; Yang *et al*., 2020; Portengen *et al*., 2022). Our results thus might indicate that the P2 appears to be a less reliable indication of gender stereotyping in the brain.

Despite the strengths of the large sample size and within-family design, the study also comes with some caveats. First, the sample consisted mainly of highly educated, White families. Therefore, the conclusions drawn from this study can only be generalized to this population. Moreover, only mixed-gender parent couples were included in this sample. This ensures that the results from this study would not be confounded by other factors than child gender, but this also limits the generalizability of findings. Third, even though pictures of parents’ own children were adjusted in the luminance range, the quality of pictures varied per family. It might be that the (lack of) effects for own *vs* unknown child are due to differences in lighting composition and/or quality of the provided pictures. Future studies might control for these factors by standardizing the picture setting for parents’ own children by having the researchers take pictures. Fourth, there were indications that the baseline correction was not completely effective in correcting for baseline drifting caused by the preceding picture stimuli. Although the biggest differences observed at baseline were found between parents’ own and unknown children, and this specific main effect was not of interest in the current study, this may still have confounded our results. By including mean baseline activity as a variable in the models that were not corrected by the preceding ERP component (i.e. the P1 and P3 models), we applied a conservative correction for carryover effects from baseline activity (Alday, 2019).

The results of this study provide several directions for future research. First, this research only examined gender stereotypes in the domain of toy preference and problem behaviors. Other domains, such as emotions, math abilities or occupational preferences, are also highly gendered (Brody and Hall, 2008; Root and Rubin, 2010; Gunderson *et al*., 2012; Wang and Degol, 2017). Future research could examine whether the results found in this study can be extended to these other gender-stereotype domains for children of different ages. Second, it would be interesting to investigate if parents’ neural responses to gender-stereotype violations by their own children are related to actual gendered parenting behaviors and emotion socialization practices (van der Pol *et al*., 2015) in the home context, since gender-stereotype violations by parents’ own children evoke different neural processes than unfamiliar children. Third, the congruence effect found in LPP amplitudes that was specific for own children warrants future research to investigate the underlying mechanisms that can explain which motivational process underlies this top-down processing strategy.

## Conclusions

This study indicated that parents differentiate in the processing of gender-stereotype violations by their own and unknown children, and between boys and girls, especially when problem behavior is described. Importantly, kinship between parent and child appeared to enhance the neural but reduce the evaluative reactions to gender-stereotype violations. Furthermore, the increased neural processing of gender-stereotype violations could partly be linked to parents’ more negative evaluations of gender-stereotype violations. Since gender-stereotype violations in the domain of problem behavior still evoke clear neural and evaluative reactions in parents, it may be especially important to make parents more aware of their stereotyped expectations of their children’s behaviors. Therefore, more research into parents’ neural processing of gendered information about their children as an underlying process of parents’ gender socialization strategies is warranted. The socialization of less rigid gender norms for child behavior can help future generations of children, and especially boys, to be able to express more gender-atypical behaviors and pursue (academic) careers often deemed gender atypical.

## Supplementary Material

nsae025_Supp

## Data Availability

The data underlying this article will be shared on reasonable request to the corresponding author.
