# Supplementary material for: Mothers’ and fathers’ neural responses toward gender-stereotype violations by their own children
Source: Soc Cogn Affect Neurosci. 2024 Mar 29;19(1):nsae025. doi: 10.1093/scan/nsae025 (PMC11026100; doi:10.1093/scan/nsae025)

**Supplementary Materials**

**Instructions Parents Photographs of Own Children**

Parents were instructed to send in a picture of their participating son and daughter with a neutral facial expression. To ensure sufficient quality and similarity, parents were instructed to upload a picture of their children wearing neutral colors and with a white background. Parents were sent an exemplar picture that was not selected for the Impression Formation Task to ensure parents knew what was expected of the photographs (see Figure S1). Pictures were visually checked by the first author CP, cropped in size, and brightness levels were adjusted to ensure that the luminance range was between 190 - 205. If the pictures were not of sufficient quality for the EEG task, parents were given more specific instructions about what needed to be adjusted (e.g., lighting, facial expression of the child, background) and requested to upload a new picture.

**Supplementary Results**

The supplementary results consist of five parts. First, we describe the steps taken and models that were compared that led to the models that are described in the main paper. The second part of the supplementary results contain the findings from the toy block in which we found no main and interaction effects of congruence on ERP mean amplitudes. Third, since the several of the findings regarding ERP mean amplitude differences during the behavior block described in the main paper are corrected for the preceding component, this section contains the uncorrected results. Fourth, since we were only interested in the congruence effects for the main paper, the fourth section describes the significant effects that were not related to congruence. Finally, we post-hoc examined the effects of congruence on N400 mean amplitudes during the toy block and behavior block. These results are described in the final Supplementary Results section.

**Model Selection Procedures**

For all models, a basic intercept model was plotted as the baseline model. First, main effects are added and compared with the intercept only model. As a second step, two-way-interactions were added and compared with the main effect model to examine model fits. If an interaction did not significantly improve the model fits, it was not added to the model. Third, three-way-interactions were added to examine if these would improve model fits. Again, if they would not improve model fits, they were omitted from the model. These steps were repeated separately for each model.

**Model Selection Toy Block**

***P1***. Adding congruence, child gender and child type as main effects significantly improved model fit from the base model (*AIC* = 33077, *χ^2^*(5) = 315.1, *p* < .001). Adding parent gender or parents’ gender attitudes about toys did not lead to an improvement of model fits (*p* = .064). Adding an interaction between congruence and child gender and congruence and child type additionally led to a significant improvement of the model (*AIC* = 32112, *χ^2^*(9) = 983.0, *p* < .001). Lastly, adding a three-way interaction between congruence, child gender, and child type significantly improved the model (*AIC* = 26130, *χ^2^*(29) = 6050.6, *p* < .001).

***N1****.* Adding congruence, child gender, and child type to the model significantly improved model fits (*AIC* = 34635, *χ^2^*(5) = 379.9, *p* < .001). When adding the main effects of parents’ gender attitudes about toys and parent gender, model fits significantly improved (*AIC* = 34633, *χ^2^*(2) = 6.1, *p* = .048). Further adding of interactions also led to convergence issues, so the final model included a main effect of congruence, child gender, child type, gender, and gender attitudes.

***P2****.*  Adding congruence, child gender, and child type to the model significantly improved model fits (*AIC* = 20629, *χ^2^*(5) 245.4, *p* < .001). Adding the main effects of gender attitudes and parent gender did not significantly improve model fits (*p* = .236). The interactions between congruence and child type and congruence and child gender significantly improved model fit (*AIC* = 18058, *χ^2^*(12) = 884.8, *p* < .001). Finally, the three way interaction between congruence, child gender, child type led to a significant improvement of model fit (*AIC* = 16985, *χ^2^*(29) = 1991.8, *p* < .001).

***P3***. Adding the main effects of congruence, child gender, and child type to the basic model significantly improved the model fit (*AIC* = 16053, *χ^2^*(12) = 1228.5, *p* < .001). Adding the main effects of parent gender or parents’ gender attitudes about toys did not significantly improve model fits thus these variables were omitted from the model (*p* = .369). Adding the interaction between congruence and child gender did not significantly improve model fit *(p* = .999*).* Adding an interaction between congruence and child type led to convergence issues and was therefore omitted. Last, the three way interaction between congruence, child gender, and child type led to a significant improvement (*AIC* = 14940, *χ^2^*(30) = 1173.0, *p* < .001).

***Late Positive Potential***. The main effects of congruence, child gender, and child type significantly improved model fits (*AIC* = 18825, *χ^2^*(5) = 269.5, *p* < .001). Adding the main effects of parent gender or parents’ gender attitudes about toys did not significantly improve the model fit (*p* = .344). Moreover, the interactions between congruence and child gender and congruence and child type led to a significant improvement of the model fit (*AIC* = 17580, *χ^2^*(9) = 1263.0, *p* < .001). Lastly, adding the three way interaction between congruence, child gender, and child type, also significantly improved model fit (*AIC* = 16157, *χ^2^*(28) = 1479.3, *p* < .001).

**Model Selection Behavior Block**

***P1****.* Adding congruence and the child variables (child gender, child type) significantly improved model fits compared to the intercept only model (*AIC* = 29968, *χ^2^*(12) = 2883.8, *p* < .001). Adding parent gender and parents’ gender attitudes did not significantly improve model fits (*p* = .600). Adding the interactions between congruence and child gender and congruence and child type also significantly improved the predictive value of the model (*AIC* = 27953, *χ^2^*(13) = 2040.7, *p* < .001). Adding a three-way interaction between congruence, child gender, and child type, and interactions between congruence and parents’ gender attitudes about behavior and congruence and parent gender significantly improved model fits (*AIC* = 25699, *χ^2^*(18) = 2289.3, *p* < .001). The final model thus included all main effects, two-way interactions between congruence and child gender/child type, and congruence and parent gender/parents’ gender attitudes about behavior, as well as an three-way interaction between congruence, child gender, and child type.

***N1***. Family as a separate random intercept was dropped for the N1 models since family explained no random variance and caused singularity issues. Relatedness was still corrected for through its nesting properties with participant ID. Adding main effects of congruence, child gender, and child type to the model significantly improved model fit compared to the intercept only model (*AIC* = 33892, *χ^2^*(5) = 483.4, *p* < .001). Moreover, adding the main effects of parent gender and parents’ gender attitudes significantly improved model fits (*AIC* = 33885, *χ^2^*(2) = 10.8, *p* = .004). Regarding the two-way interactions, congruence and child gender/child type and congruence and parents’ gender attitudes and parent gender significantly improved the model’s fit (*AIC* = 29494, *χ^2^*(23) = 4437.2, *p* < .001). Adding a three-way interaction between congruence, child gender, and child type additionally improved model fits (*AIC* = 27717, *χ^2^*(15) = 1762.0, *p* < .001).

***P2***. Adding all stimulus-related main effects led to a significant improvement of the base model child variables: *AIC* = 18985*, χ^2^*(12) = 1584.6, *p* < .001) but adding a main effect of parent gender and parents’ gender attitudes did not significantly improve model fit (*p* = .836). Adding interactions between congruence and child gender and child type also significantly improved the model fit (*AIC* = 17819, *χ^2^*(13) = 1191.8, *p* < .001), as did the interactions between congruence and parents’ gender attitudes (*AIC* = 17817, *χ^2^*(2) = 6.0, *p* = .050). The interaction between congruence and parent gender led to convergence issues and was thus omitted. Finally, a three-way interaction between congruence, child gender, and child type was added, which again improved model fit (*AIC* = 16859, *χ^2^*(17) = 991.8, *p* < .001). Additionally, a three-way interaction between congruence, child type, and parents’ gender attitudes about behavior was added, which also improved the model (*AIC* = 16852, *χ^2^*(2) = 11.12, *p* = .004) but adding a three-way interaction between congruence, child gender, and parents’ gender attitudes did not (*p* = .744).

***P3****.* Adding the main effects of congruence, child gender, and child type significantly improved model fit compared to the intercept only model (*AIC* = 15955, *χ^2^*(12) = 287.0, *p* < .001), but the main effects of parent gender and parents’ gender attitudes did not significantly improve model fit (*p* = .772). Adding interactions between congruence and child gender, and congruence and child type also significantly improved model fit (*AIC* = 15562, *χ^2^*(13) = 419.2, *p* < .001), as did the three-way interaction between these variables (*AIC* = 14660, *χ^2^*(17) = 935.7, *p* < .001).

***Late Positive Potential***. Lastly for the LPP model, the main effects of congruence, child gender, and child type significantly improved model fit (*AIC* = 17272, *χ^2^*(11) = 1092.8, *p* < .001) but adding the main effect of parents’ gender attitudes and parent gender did not. Adding the two-way interactions between congruence and child gender and congruence and child type also significantly improved model fit (*AIC* = 16702, *χ^2^*(13) = 595.7, *p* < .001). Finally, the three-way interaction between congruence, child gender, and child type also significantly decreased AIC values for the LPP mean amplitude model (*AIC* = 16210, *χ^2^*(18) = 527.8, *p* < .001).

**Parents’ Neural Responses Towards the Violation of Gender Stereotypes about Toys**

***N1****.* The final model included congruence, child gender, child type, parent gender, and gender attitudes as main effects. Significant main effects were found for parent gender (*β* = -.12, t(135) = -2.28, *p* = .024) and child type (*β* = .08, *t*(135) = 4.51, *p* < .001). Mothers had more negative (stronger) N1 mean amplitudes regardless of which type of stimuli was shown. A stronger N1 effect (more negative amplitudes) was observed towards unknown children than own children.

***P2****.* The final model included a main effect of congruence, child type and child gender, as well as a three-way interaction between these three variables. A main effect of child type was found for P2 amplitudes (*β* = .14, *t*(135) = 5.27, *p* < .001). Parents’ P2 mean amplitudes were more positive towards their own than unknown children. None of the other main effects were significant (smallest *p*-value = .102), nor did the two-way and three-way interactions yield a significant result (*p*s > .103).

***P3****.* Similar to the previous components the final model included a main effect of congruence, child gender, and child type, as well as two- and three-way interactions between these three variables The main effect of congruence was not significant (*p* = .157) nor were the interactions between congruence and the other variables significant (lowest *p*-value = .091). No other main effects were significant (*p*s > .313).

***Late Positive Potential****.* Again, the final model included a main effect of congruence, child gender, and child type, as well as the two- an three-way interactions between these variables. There was no significant main effect of congruence on LPP mean amplitudes (*p* = .375), nor were significant interaction effects found (lowest *p*-value = .222). Again, a main effect for child type was found (*β* = .11, *t*(135) = 4.25, *p* < .001). Parents’ LPP amplitudes were larger during trials that included their own children compared to trials with unknown children.

**Parents’ Neural Responses Towards the Violation of Gender Stereotypes about Behavior Uncorrected Results**

***N1****.* A main effect was found for congruence (*β* = .08, *t*(135) = 3.24, *p* = .001), child type (*β* = .12, *t*(135) = 4.96, *p* < .001), and child gender (*β* = .05, *t*(135) = 3.15, *p* = .002). N1 mean amplitudes were more negative towards congruent trials than incongruent trials, toward unknown children than parents’ own children, and toward boys than girls. Moreover, the two-way interactions between congruence and child gender (*β* = -.07, *t*(135) = -3.54, *p* < .001) and between congruence and child type (*β* = -.05, *t*(135) = -2.05, *p* = .043) were significant but these were subsumed under a significant three-way interaction between congruence, child gender, and child type (*β =* .05, *t*(135) = 2.16, *p* = .034). Decomposing this three-way interaction effect revealed that for unknown children, there was a significant interaction between congruence and child gender (*β* = -.06, *t*(135) = -2.54, *p* = .001) but this interaction was not found for trials that included parents’ own children (*p* = .855). Running the models separately for unknown boys and unknown girls revealed that parents’ N1 mean amplitudes were larger (more negative) toward gender-stereotype-confirming than gender stereotype-violating boys (*β* = .33, *t*(135) = 2.56, *p* = .012) but no effect of congruence was observed for girls (*p* = .132).

***P2****.* A significant main effect was found for congruence (*β* = .07, *t*(135) = 2.52 *p* = .013) and child type (*β* = .08, *t*(135) = 2.22, *p* = .028). P2 amplitudes were more positive during incongruent trials than congruent trials and toward parents’ own children compared to unknown children. Additionally, a significant main effect of parents’ gender attitudes about behavior was found (*β* = -.21, *t*(135) = -2.52, *p* = .013). P2 amplitudes were weaker when parents held more traditional gender attitudes Moreover, a significant interaction was found between congruence and child gender (*β* = -.08, *t*(135) = -3.06, *p* = .003). Running the model separately for trials with boys and trials with girls revealed that for boys, parents P2 amplitudes were larger during incongruent trials than during congruent trials (*β* = .06, *t*(189) = 2.00, *p* = .047). For girls, there was no main effect of congruence (*p* = .378). Lastly, interactions were found between child gender and child type (*β* = -.06, *t*(135) = -2.12, *p* = .036) child type and parents’ gender attitudes about behavior (*β* = .12, *t*(135) = 2.97, *p* = .004) (see Figure S3).

***Late Positive Potential****.* A significant main effect was found for child type (*β* = .11, *t*(135) = 4.84, *p* < .001). LPP mean amplitudes were larger during incongruent trials and during trials that included parents’ own children than unknown children. Moreover, a significant interaction was found between congruence and child gender (*β* = -.05, *t*(135) = -2.11, *p* = .036). Subsequent analyses revealed no significant effects of congruence or child gender in the post-hoc comparisons.

**Findings in the Neural Components Corrected for the Preceding Component Unrelated to Congruence**

***N1****.* With regard to N1 amplitudes, a significant main effect of parents’ gender attitudes on N1 amplitudes was found; parents’ N1 responses towards congruent and incongruent child-behavior combinations were stronger when parents held more traditional gendered attitudes about behavior (*β* = -.07, *t*(135) = -2.37, *p* = .020). Moreover, N1 mean amplitudes were significantly stronger (more negative) toward unknown children compared to parents’ own children (*β* = .03, *t*(135) = 2.45, *p* = .015).

***P2****.* A significant interaction was found between child type and parents’ gender attitudes about behavior (*β* = .06, *t*(135) = 2.71, *p* = .007). When parents held stronger gender attitudes about behavior (i.e., rated congruent trials as more appropriate than incongruent trials), parents’ P2 amplitudes towards unknown children decreased, but parents’ gender attitudes about behavior did not affect parents’ P2 mean amplitudes towards their own children, see Figure S3.

**Post-hoc Examination of Congruence Effects in N400 Mean Amplitudes**

The effects of congruence for the N400 mean amplitudes were post-hoc examined for several reasons. First, we based our ERP selection on previous studies that examined congruence effects with a similar task design (e.g., (Portengen et al., 2022)) that did not include the N400 in their ERP component selection. Second, the N400 has mainly shown effects in response to linguistic priming rather than in response to face-word combinations (e.g., (Franklin et al., 2007; Rodríguez-Gómez et al., 2020). Third, the N400 effect can be obscured when LPP responses are measured (Franklin et al., 2007). However, since the N400 has also been found to be modulated in response to stereotyped information (e.g., (White et al., 2009), we post-hoc examined the effect of congruence in N400 mean amplitudes.

In order to do this, N400 mean amplitudes were exported from the fronto-central electrodes (Fz, Cz, Pz, FC1, FC2, C3, C4) in the 300-500ms time window (in accordance with White et al. (2009). The analyses revealed no effect of congruence on N400 mean amplitudes in the toy or the behavior block, nor were any of the interactions significant.

The final N400 mean amplitude models included a main effect of congruence, child gender, and child type, as well as an interaction between congruence and child gender and congruence and child type. For the N400 mean amplitude model in the behavior block, an additional higher-order interaction was added between child gender, child type, and congruence.

There was no significant main effect of congruence in the N400 toy block model (*β* = .03, *t*(136) = 1.00, = .317). Moreover, no significant interaction was found between congruence and child gender (*β* = -.01, *t*(136) = -0.48, *p* = .633) or congruence and child type (*β* = -.01, *t*(136) = -1.13, *p* = .846). Similarly, no main effect of congruence was found in the N400 behavior block model (*β* = -.01, *t*(135) = -1.03, *p* = .695) nor were the interaction between congruence and child gender or congruence and child type significant (child gender: *β* = .02, *t*(135) = 0.89, *p* = 376; child type: *β* = 05, t(135) = 1.76 *p* = .080). Last, no significant three-way interaction emerged between congruence, child gender, and child type (*β* = -.07, *t*(135) = -1.57, *p* = .120).

**References**

Franklin, M. S., Dien, J., Neely, J. H., Huber, E., & Waterson, L. D. (2007). Semantic priming modulates the n400, n300, and n400rp. *Clinical Neurophysiology*, *118*(5), 1053-1068. https://doi.org/10.1016/j.clinph.2007.01.012

Portengen, C. M., Huffmeijer, R., van Baar, A. L., & Endendijk, J. J. (2022). Measuring the neural correlates of the violation of social expectations: A comparison of two experimental tasks. *Social Neuroscience*, *17*(1), 58-72. https://doi.org/10.1080/17470919.2022.2032327

Rodríguez-Gómez, P., Romero-Ferreiro, V., Pozo, M. A., Hinojosa, J. A., & Moreno, E. M. (2020). Facing stereotypes: Erp responses to male and female faces after gender-stereotyped statements. *Social Cognitive and Affective Neuroscience*, *15*(9), 928-940. https://doi.org/10.1093/scan/nsaa117

White, K. R., Crites, S. L., Jr., Taylor, J. H., & Corral, G. (2009). Wait, what? Assessing stereotype incongruities using the n400 erp component. *Social Cognitive and Affective Neuroscience*, *4*(2), 191-198. https://doi.org/10.1093/scan/nsp004

**Table S1.**

*Results from the Multilevel Models Examining Congruence Effects on ERP Mean Amplitudes During Toy Trials.*

| **P1** | *b* | *β* | *SE* | *t*(df) | *p* |
| --- | --- | --- | --- | --- | --- |
| Congruence | 0.15 | .02 | .09 | 1.65 (135) | .101 |
| Child gender | 0.40 | .07 | .03 | 1.88 (135) | .063 |
| Child type | 0.22^*^ | .04 | .11 | 3.90 (135) | <.001 |
| Congruence*child gender | -0.33^*^ | -.05 | .14 | -2.35 (135) | .020 |
| **N1** | *b* | *β* | *SE* | *t*(df) | *p* |
| Congruence | -0.01 | -.001 | .06 | -0.49 (136) | .883 |
| Child gender | 0.13 | .02 | .08 | 1.53 (135) | .129 |
| Child type | 0.51^*^ | .08 | .11 | 4.51 (135) | <.001 |
| Gender | -0.77^*^ | -.12 | .34 | -2.28 (135) | .024 |
| Gender attitudes about toys | -0.04 | -.02 | .12 | -0.35 (135) | .724 |
| **P2** | *b* | *β* | *SE* | *t*(df) | *p* |
| Congruence | 0.23 | .04 | .14 | 1.65 (135) | .104 |
| Child gender | 0.14 | .02 | .12 | 1.16 (135) | .248 |
| Child type | 0.82^*^ | .14 | .16 | 5.27 (135) | <.001 |
| Congruence*child gender | -0.18 | -.03 | .17 | -1.11 (135) | .267 |
| Congruence*child type | -0.34 | -.05 | .21 | -1.64 (135) | .103 |
| Child gender*child type | -0.15 | -.02 | .36 | 0.83 (135) | .409 |
| Congruence*child gender*child type | 0.04 | .01 | .26 | 0.16 (135) | .877 |
| **P3** | *b* | *β* | *SE* | *t(*df) | *p* |
| Congruence | 0.30 | .06 | .21 | 1.42 (135) | .158 |
| Child gender | 0.10 | .02 | .13 | -0.73 (135) | .470 |
| Child type | -0.13 | -.03 | .12 | -1.01 (135) | .313 |
| Congruence*child gender | -0.33 | -.06 | .19 | -1.71 (135) | .091 |
| Congruence*child type | -0.28 | -.05 | .26 | -1.07 (135) | .289 |
| Child gender*child type | -0.16 | -.03 | .16 | -0.99 (135) | .322 |
| Congruence*child gender*child type | 0.44 | .06 | .29 | 1.50 (135) | .136 |
| **Late Positive Potential** | *b* | *β* | *SE* | *t*(df) | *p* |
| Congruence | 0.20 | .03 | .22 | 0.89 (135) | .375 |
| Child gender | 0.04 | .005 | .19 | -0.19 (135) | .848 |
| Child type | 0.84^*^ | .11 | .20 | 4.25 (135) | <.001 |
| Congruence*child gender | -0.003 | .0004 | .23 | -0.01 (135) | .989 |
| Congruence*child type | 0.23 | -.03 | .28 | -0.84 (135) | .402 |
| Child gender*child type | -0.30 | -.03 | .24 | -1.23 (135) | .222 |
| Congruence*child gender*child type | 0.28 | .02 | .34 | 0.82 (135) | .416 |

*Note*. GAT = gender attitudes about toys. Child type refers to the difference between parents’ own children and unknown children. Congruent, boy, unknown child, and father were the reference categories for congruence, child gender, child type, and parent gender, respectively.

The asterisk indicates significant effects with *p* < .05.

**Table S2.**

*Results from the Multilevel Models Examining Congruence Effects on ERP Mean Amplitudes During Behavior Trials Uncorrected for the Preceding Component.*

| **N1** | *b* | *β* | *SE* | *t*(df) | *p* |
| --- | --- | --- | --- | --- | --- |
| Congruence | 0.50^*^ | .08 | .15 | 3.24 (135) | .001 |
| Child gender | 0.31^*^ | .05 | .10 | 3.15 (135) | .002 |
| Child type | 0.79^*^ | .12 | .16 | 4.96 (135) | <.001 |
| Parent gender | -0.34 | -.05 | .33 | -1.05 (135) | .299 |
| GAB | -0.38 | -.08 | .23 | -1.64 (135) | .105 |
| Congruence*GAB | -0.16 | -.03 | .08 | -1.97 (135) | .051 |
| Congruence*child gender | -0.51^*^ | -.07 | .14 | -3.54 (135) | .001 |
| Congruence*child type | -0.36 | -.05 | .18 | -2.05 (135) | .043 |
| Congruence*parent gender | -0.10 | -.01 | .12 | -0.88 (135) | .381 |
| Child gender*child type | -0.40^*^ | -.05 | .17 | -2.32 (135) | .022 |
| Congruence*child gender*child type | 0.55^*^ | .05 | .25 | 2.16 (135) | .033 |
| **P2** | *b* | *β* | *SE* | *t*(df) | *p* |
| Congruence | 0.39^*^ | .07 | .15 | 2.52 (135) | .013 |
| Child gender | 0.19 | .03 | .10 | 1.79 (135) | .075 |
| Child type | 0.43^*^ | .08 | .19 | 2.22 (135) | .028 |
| GAB | -0.80^*^ | -.21 | .32 | -2.52 (135) | .013 |
| Congruence*GAB | -0.11 | -.03 | .13 | -0.88 (135) | .077 |
| Congruence*child gender | -0.45^*^ | -.07 | .15 | -3.06 (135) | .003 |
| Congruence*child type | -0.28 | -.04 | .23 | -1.19 (135) | .236 |
| Child gender*child type | -0.32^*^ | -.05 | .15 | -2.12 (135) | .036 |
| Child type*GAB | 0.52^*^ | .12 | .17 | 2.98 (135) | .003 |
| Congruence*child gender*child type | 0.42 | .05 | .25 | 1.65 (135) | .102 |
| Congruence*child type*GAB | -0.02 | -.004 | .22 | -0.10 (135) | .926 |
| **Late Positive Potential** | *b* | *β* | *SE* | *t*(df) | *p* |
| Congruence | 0.27 | .04 | .15 | 1.82 (135) | .070 |
| Child gender | 0.16 | .02 | .14 | 1.16 (135) | .248 |
| Child type | 0.84^*^ | .11 | .17 | 4.84 (135) | <.001 |
| Congruence*child gender | -0.40^*^ | -.05 | .19 | -2.12 (135) | .036 |
| Congruence*child type | 0.03 | .003 | .18 | 0.16 (135) | .877 |
| Child gender*child type | -0.12 | -.01 | .22 | -0.55 (135) | .586 |
| Congruence*child gender*child type | 0.05 | .004 | .31 | 0.16 (135) | .871 |
|  |  |  |  |  |  |

*Note*. GAB = gender attitudes about behavior. Congruent, boy, unknown child, and father were the reference categories for congruence, child gender, child type, and parent gender, respectively.

The asterisk indicates significant effects with *p* < .05.

**Table S3.**

*Zero-Order Correlations Between the ERP Mean Amplitudes During the Behavior Block and Children’s Parent-Reported Internalizing and Externalizing Behaviors.*

|  | Dau Internalizing | Son Internalizing | Dau Externalizing | Son Externalizing |
| --- | --- | --- | --- | --- |
| P1 Pz boy con | -0.024 | 0.055 | 0.153 | 0.080 |
| P1 Pz boy incon | -0.086 | 0.026 | 0.167 | 0.022 |
| P1 Pz girl con | -0.071 | 0.049 | 0.146 | 0.102 |
| P1 Pz girl incon | -0.057 | 0.074 | 0.138 | 0.098 |
| P1 Pz son con | -0.027 | 0.054 | 0.124 | 0.146 |
| P1 Pz son incon | -0.006 | 0.023 | 0.178 | 0.100 |
| P1 Pz dau con | -0.054 | 0.020 | 0.103 | 0.077 |
| P1 Pz dau incon | -0.058 | 0.077 | 0.150 | 0.050 |
| P1 P3 boy con | -0.025 | 0.113 | 0.187 | 0.087 |
| P1 P3 boy incon | -0.073 | 0.092 | 0.154 | 0.045 |
| P1 P3 girl con | -0.072 | 0.118 | 0.157 | 0.080 |
| P1 P3 girl incon | -0.034 | 0.136 | 0.160 | 0.091 |
| P1 P3 son con | 0.009 | 0.160 | 0.206 | 0.199 |
| P1 P3 son incon | 0.025 | 0.116 | 0.186 | 0.122 |
| P1 P3 dau con | -0.013 | 0.148 | 0.182 | 0.185 |
| P1 P3 dau incon | -0.046 | 0.209 | 0.179 | 0.075 |
| P1 P4 boy con | -0.078 | 0.077 | 0.161 | 0.071 |
| P1 P4 boy incon | -0.106 | 0.049 | 0.157 | 0.044 |
| P1 P4 girl con | -0.095 | 0.064 | 0.159 | 0.059 |
| P1 P4 girl incon | -0.066 | 0.090 | 0.184 | 0.085 |
| P1 P4 son con | -0.005 | 0.117 | 0.185 | 0.160 |
| P1 P4 son incon | -0.057 | 0.040 | 0.159 | 0.072 |
| P1 P4 dau con | -0.057 | 0.045 | 0.152 | 0.044 |
| P1 P4 dau incon | -0.023 | 0.074 | 0.165 | 0.064 |
| P1 PO3 boy con | -0.031 | 0.104 | 0.176 | 0.076 |
| P1 PO3 boy incon | -0.056 | 0.116 | 0.164 | 0.002 |
| P1 PO3 girl con | -0.068 | 0.085 | 0.139 | 0.034 |
| P1 PO3 girl incon | -0.022 | 0.121 | 0.163 | 0.074 |
| P1 PO3 son con | 0.012 | 0.132 | 0.131 | 0.172 |
| P1 PO3 son incon | 0.025 | 0.027 | 0.146 | 0.139 |
| P1 PO3 dau con | -0.012 | 0.115 | 0.156 | 0.104 |
| P1 PO3 dau incon | -0.032 | 0.162 | 0.150 | 0.069 |
| P1 PO4 boy con | -0.060 | 0.051 | 0.177 | 0.078 |
| P1 PO4 boy incon | -0.064 | 0.049 | 0.177 | 0.036 |
| P1 PO4 girl con | -0.101 | 0.045 | 0.140 | 0.040 |
| P1 PO4 girl incon | -0.056 | 0.100 | 0.159 | 0.075 |
| P1 PO4 son con | -0.041 | 0.094 | 0.175 | 0.115 |
| P1 PO4 son incon | 0.001 | 0.027 | 0.199 | 0.118 |
| P1 PO4 dau con | -0.045 | 0.016 | 0.165 | 0.043 |
| P1 PO4 dau incon | -0.018 | 0.089 | 0.179 | 0.079 |
| P1 O1 boy con | -0.073 | 0.126 | 0.150 | 0.075 |
| P1 O1 boy incon | -0.085 | 0.100 | 0.113 | -0.023 |
| P1 O1 girl con | -0.099 | 0.088 | 0.102 | 0.036 |
| P1 O1 girl incon | -0.072 | 0.135 | 0.099 | 0.036 |
| P1 O1 son con | -0.044 | 0.144 | 0.106 | 0.078 |
| P1 O1 son incon | 0.005 | 0.046 | 0.100 | 0.121 |
| P1 O1 dau con | -0.063 | 0.086 | 0.080 | 0.020 |
| P1 O1 dau incon | -0.047 | 0.138 | 0.114 | 0.047 |
| P1 O2 boy con | -0.088 | 0.051 | 0.185 | 0.084 |
| P1 O2 boy incon | -0.112 | 0.042 | 0.153 | -0.017 |
| P1 O2 girl con | -0.118 | 0.073 | 0.189 | 0.050 |
| P1 O2 girl incon | -0.068 | 0.095 | 0.135 | 0.029 |
| P1 O2 son con | -0.044 | 0.085 | 0.182 | 0.076 |
| P1 O2 son incon | -0.008 | 0.048 | 0.209 | 0.108 |
| P1 O2 dau con | -0.100 | 0.035 | 0.156 | 0.011 |
| P1 O2 dau incon | -0.005 | 0.090 | 0.186 | 0.052 |
| P1 Oz boy con | -0.086 | 0.080 | 0.176 | 0.068 |
| P1 Oz boy incon | -0.101 | 0.068 | 0.146 | -0.016 |
| P1 Oz girl con | -0.094 | 0.045 | 0.171 | 0.045 |
| P1 Oz girl incon | -0.058 | 0.110 | 0.133 | 0.028 |
| P1 Oz son con | -0.062 | 0.126 | 0.126 | 0.046 |
| P1 Oz son incon | 0.008 | 0.026 | 0.141 | 0.102 |
| P1 Oz dau con | -0.093 | 0.020 | 0.113 | 0.020 |
| P1 Oz dau incon | -0.044 | 0.073 | 0.156 | 0.068 |
| P3 FC1 boy con | 0.101 | -0.058 | -0.051 | 0.078 |
| P3 FC1 boy incon | -0.022 | -0.143 | 0.027 | 0.156 |
| P3 FC1 girl con | 0.088 | 0.045 | -0.028 | 0.186 |
| P3 FC1 girl incon | 0.008 | -0.027 | -0.021 | 0.192 |
| P3 FC1 son con | -0.089 | 0.063 | 0.038 | 0.073 |
| P3 FC1 son incon | -0.072 | -0.027 | -0.045 | 0.046 |
| P3 FC1 dau con | -0.050 | -0.053 | 0.094 | 0.086 |
| P3 FC1 dau incon | 0.027 | 0.028 | 0.122 | 0.090 |
| P3 FC2 boy con | 0.000 | -0.156 | -0.028 | 0.141 |
| P3 FC2 boy incon | -0.100 | -0.055 | -0.009 | 0.187 |
| P3 FC2 girl con | 0.021 | 0.002 | -0.034 | 0.171 |
| P3 FC2 girl incon | -0.003 | -0.090 | -0.085 | 0.040 |
| P3 FC2 son con | -0.073 | 0.032 | -0.037 | 0.091 |
| P3 FC2 son incon | -0.014 | -0.038 | -0.167 | 0.006 |
| P3 FC2 dau con | -0.057 | -0.104 | 0.075 | 0.058 |
| P3 FC2 dau incon | 0.025 | 0.013 | 0.133 | 0.060 |
| P3 Fz boy con | 0.071 | -0.053 | -0.164 | 0.019 |
| P3 Fz boy incon | 0.031 | -0.082 | -0.123 | 0.096 |
| P3 Fz girl con | 0.176 | 0.010 | -0.190 | 0.069 |
| P3 Fz girl incon | 0.090 | -0.046 | -0.169 | 0.063 |
| P3 Fz son con | -0.040 | -0.019 | -0.157 | -0.029 |
| P3 Fz son incon | -0.018 | -0.015 | -0.187 | -0.042 |
| P3 Fz dau con | -0.027 | -0.093 | -0.072 | -0.001 |
| P3 Fz dau incon | 0.064 | 0.035 | -0.030 | 0.119 |
| P3 Cz boy con | -0.045 | -0.017 | 0.067 | 0.182 |
| P3 Cz boy incon | -0.182 | -0.051 | 0.125 | 0.089 |
| P3 Cz girl con | -0.031 | 0.092 | 0.095 | 0.192 |
| P3 Cz girl incon | -0.047 | 0.019 | 0.066 | 0.121 |
| P3 Cz son con | -0.100 | 0.068 | 0.069 | 0.166 |
| P3 Cz son incon | -0.035 | 0.045 | 0.020 | 0.068 |
| P3 Cz dau con | -0.069 | -0.016 | 0.103 | 0.104 |
| P3 Cz dau incon | -0.020 | 0.002 | 0.163 | 0.064 |
| LPP P3 boy con | -0.101 | -0.007 | 0.155 | -0.007 |
| LPP P3 boy incon | -0.094 | 0.013 | 0.109 | -0.028 |
| LPP P3 girl con | -0.070 | 0.023 | 0.127 | -0.005 |
| LPP P3 girl incon | -0.077 | 0.036 | 0.144 | -0.021 |
| LPP P3 son con | -0.035 | 0.034 | 0.177 | 0.067 |
| LPP P3 son incon | -0.018 | -0.003 | 0.096 | 0.018 |
| LPP P3 dau con | -0.065 | -0.024 | 0.098 | -0.005 |
| LPP P3 dau incon | -0.070 | 0.031 | 0.110 | -0.065 |
| LPP P4 boy con | -0.094 | -0.008 | 0.136 | 0.048 |
| LPP P4 boy incon | -0.107 | -0.075 | 0.139 | 0.059 |
| LPP P4 girl con | -0.088 | -0.040 | 0.122 | 0.049 |
| LPP P4 girl incon | -0.067 | -0.002 | 0.088 | 0.059 |
| LPP P4 son con | 0.022 | 0.011 | 0.095 | 0.100 |
| LPP P4 son incon | -0.015 | -0.019 | 0.101 | 0.102 |
| LPP P4 dau con | -0.088 | -0.123 | 0.049 | 0.063 |
| LPP P4 dau incon | -0.017 | -0.072 | 0.096 | 0.036 |
| LPP PO3 boy con | -0.112 | 0.046 | 0.186 | -0.010 |
| LPP PO3 boy incon | -0.065 | 0.046 | 0.134 | -0.010 |
| LPP PO3 girl con | -0.112 | 0.011 | 0.139 | -0.014 |
| LPP PO3 girl incon | -0.105 | 0.040 | 0.155 | 0.023 |
| LPP PO3 son con | -0.008 | 0.065 | 0.109 | 0.050 |
| LPP PO3 son incon | -0.015 | 0.053 | 0.096 | 0.050 |
| LPP PO3 dau con | -0.035 | 0.017 | 0.094 | 0.013 |
| LPP PO3 dau incon | -0.020 | 0.067 | 0.089 | -0.050 |
| LPP PO4 boy con | -0.124 | -0.019 | 0.126 | 0.090 |
| LPP PO4 boy incon | -0.100 | -0.027 | 0.147 | 0.044 |
| LPP PO4 girl con | -0.119 | -0.027 | 0.091 | 0.033 |
| LPP PO4 girl incon | -0.112 | 0.045 | 0.099 | 0.086 |
| LPP PO4 son con | 0.004 | 0.033 | 0.085 | 0.064 |
| LPP PO4 son incon | -0.001 | 0.003 | 0.133 | 0.146 |
| LPP PO4 dau con | -0.092 | -0.105 | 0.041 | 0.045 |
| LPP PO4 dau incon | -0.036 | -0.023 | 0.091 | 0.058 |

*Note*. con = congruent. incon = incongruent. dau = daughter. LPP = late positive potential.

**Figure S1.**

*EXEMPLAR PICTURE USED FOR THE INSTRUCTIONS FOR THE PICTURE OF THE OWN CHILD WITH NEUTRAL FACIAL EXPRESSION.*


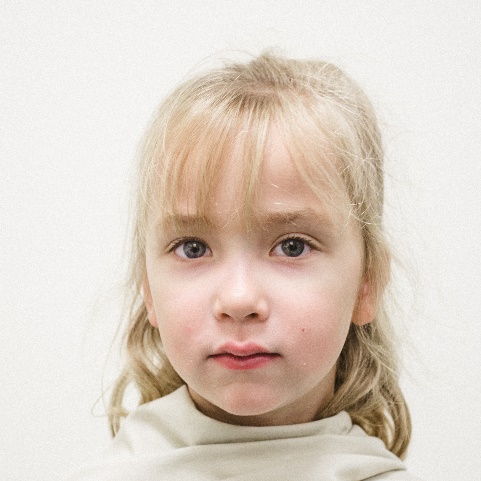


**Figure S2.**

*GRAND AVERAGE WAVEFORMS PER CONDITION, SEPARATE FOR THE TOY AND BEHAVIOR BLOCK.*


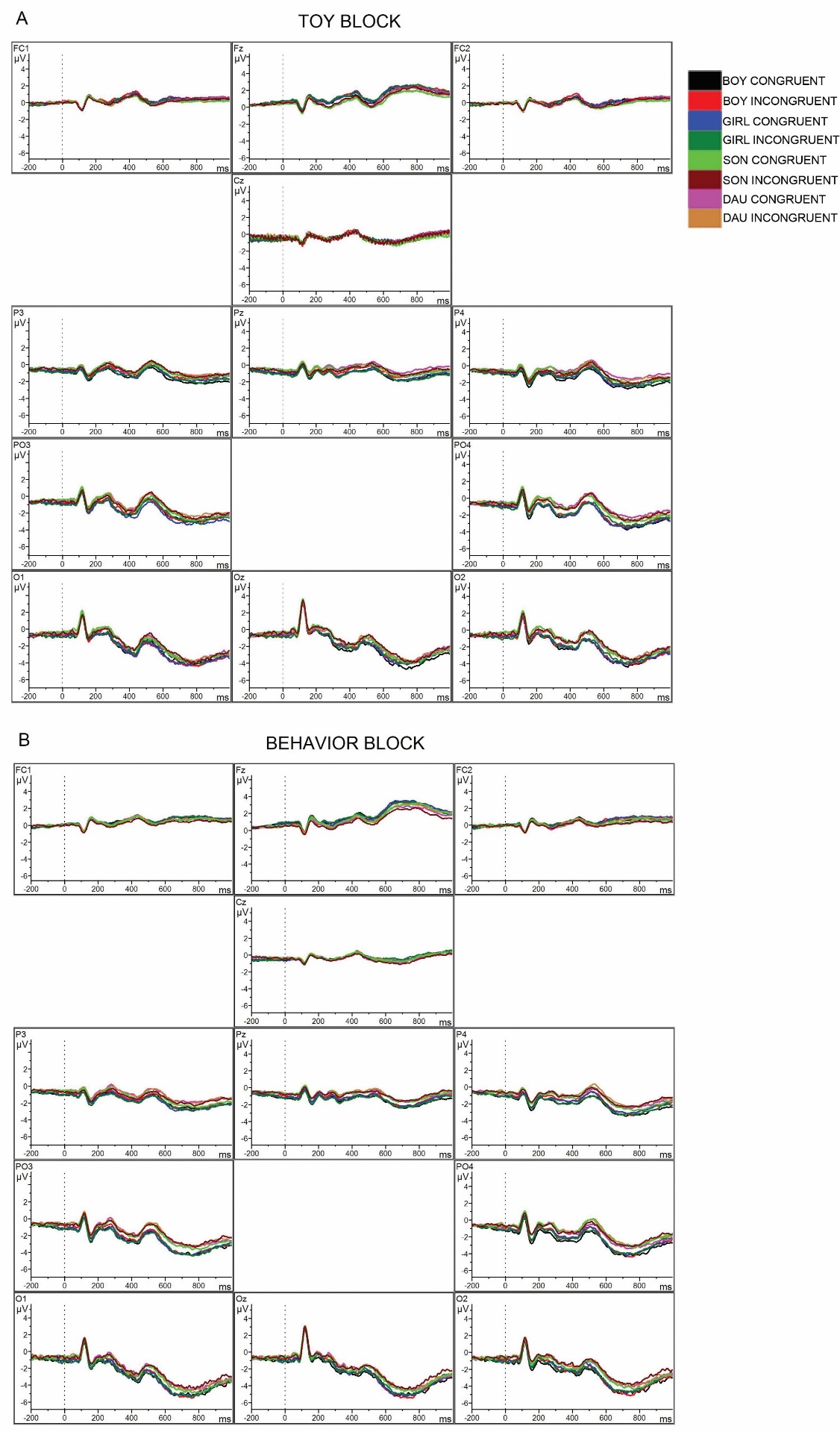


**Figure S3.**

*EFFECT OF PARENTS’ GENDER ATTITUDES ABOUT CHILD PROBLEM BEHAVIOR ON PARENTS’ P2 MEAN AMPLITUDES, SEPARATE FOR UNKNOWN CHILDREN (BLACK LINE) AND THEIR OWN CHILDREN (GREY LINE).*


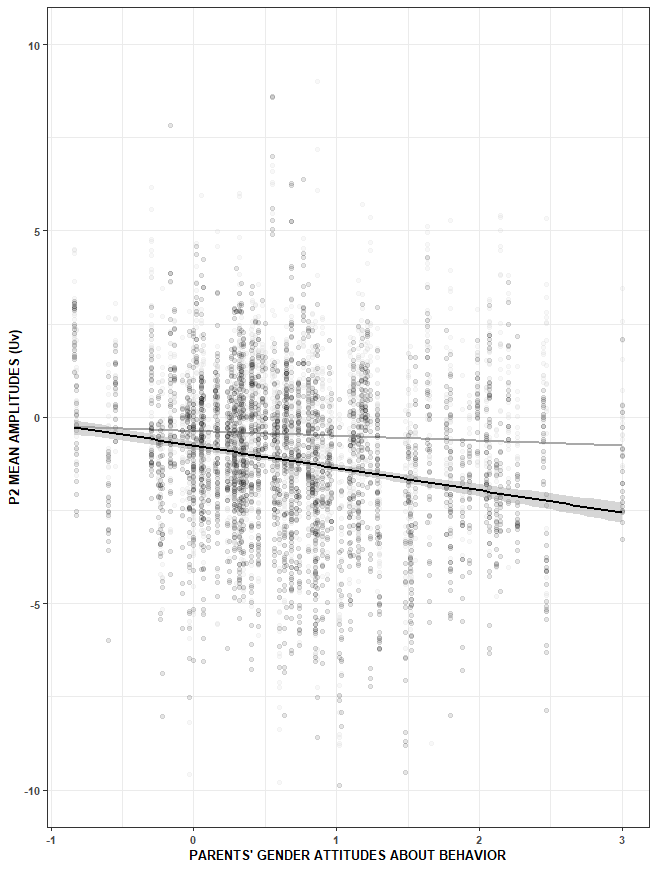

Supplement: nsae025_Supp [file nsae025_supp.zip › scan-23-019-File006.docx]
